# The Stringent Response Inhibits 70S Ribosome Formation in *Staphylococcus aureus* by Impeding GTPase-Ribosome Interactions

**DOI:** 10.1128/mBio.02679-21

**Published:** 2021-11-09

**Authors:** Daniel J. Bennison, Jose A. Nakamoto, Timothy D. Craggs, Pohl Milón, John B. Rafferty, Rebecca M. Corrigan

**Affiliations:** a The Florey Institute, Department of Molecular Biology and Biotechnology, University of Sheffield, Sheffield, United Kingdom; b Laboratory of Applied Biophysics and Biochemistry, Centre for Research and Innovation, Health Sciences Faculty, Universidad Peruana de Ciencias Aplicadas (UPC), Lima, Peru; c Sheffield Institute for Nucleic Acids, Department of Chemistry, University of Sheffield, Sheffield, United Kingdom; d Department of Molecular Biology and Biotechnology, University of Sheffield, Sheffield, United Kingdom; Massachusetts Institute of Technology

**Keywords:** GTPase, *Staphylococcus aureus*, ppGpp, ribosomes, stringent response

## Abstract

During nutrient limitation, bacteria produce the alarmones (p)ppGpp as effectors of a stress signaling network termed the stringent response. RsgA, RbgA, Era, and HflX are four ribosome-associated GTPases (RA-GTPases) that bind to (p)ppGpp in Staphylococcus aureus. These enzymes are cofactors in ribosome assembly, where they cycle between the ON (GTP-bound) and OFF (GDP-bound) ribosome-associated states. Entry into the OFF state occurs upon hydrolysis of GTP, with GTPase activity increasing substantially upon ribosome association. When bound to (p)ppGpp, GTPase activity is inhibited, reducing 70S ribosome assembly and growth. Here, we determine how (p)ppGpp impacts RA-GTPase-ribosome interactions. We show that RA-GTPases preferentially bind to 5′-diphosphate-containing nucleotides GDP and ppGpp over GTP, which is likely exploited as a regulatory mechanism within the cell to shut down ribosome biogenesis during stress. Stopped-flow fluorescence and association assays reveal that when bound to (p)ppGpp, the association of RA-GTPases to ribosomal subunits is destabilized, both *in vitro* and within bacterial cells. Consistently, structural analysis of the ppGpp-bound RA-GTPase RsgA reveals an OFF-state conformation similar to the GDP-bound state, with the G2/switch I loop adopting a conformation incompatible with ribosome association. Altogether, we highlight (p)ppGpp-mediated inhibition of RA-GTPases as a major mechanism of stringent response-mediated ribosome assembly and growth control.

## INTRODUCTION

**FIG 1:**
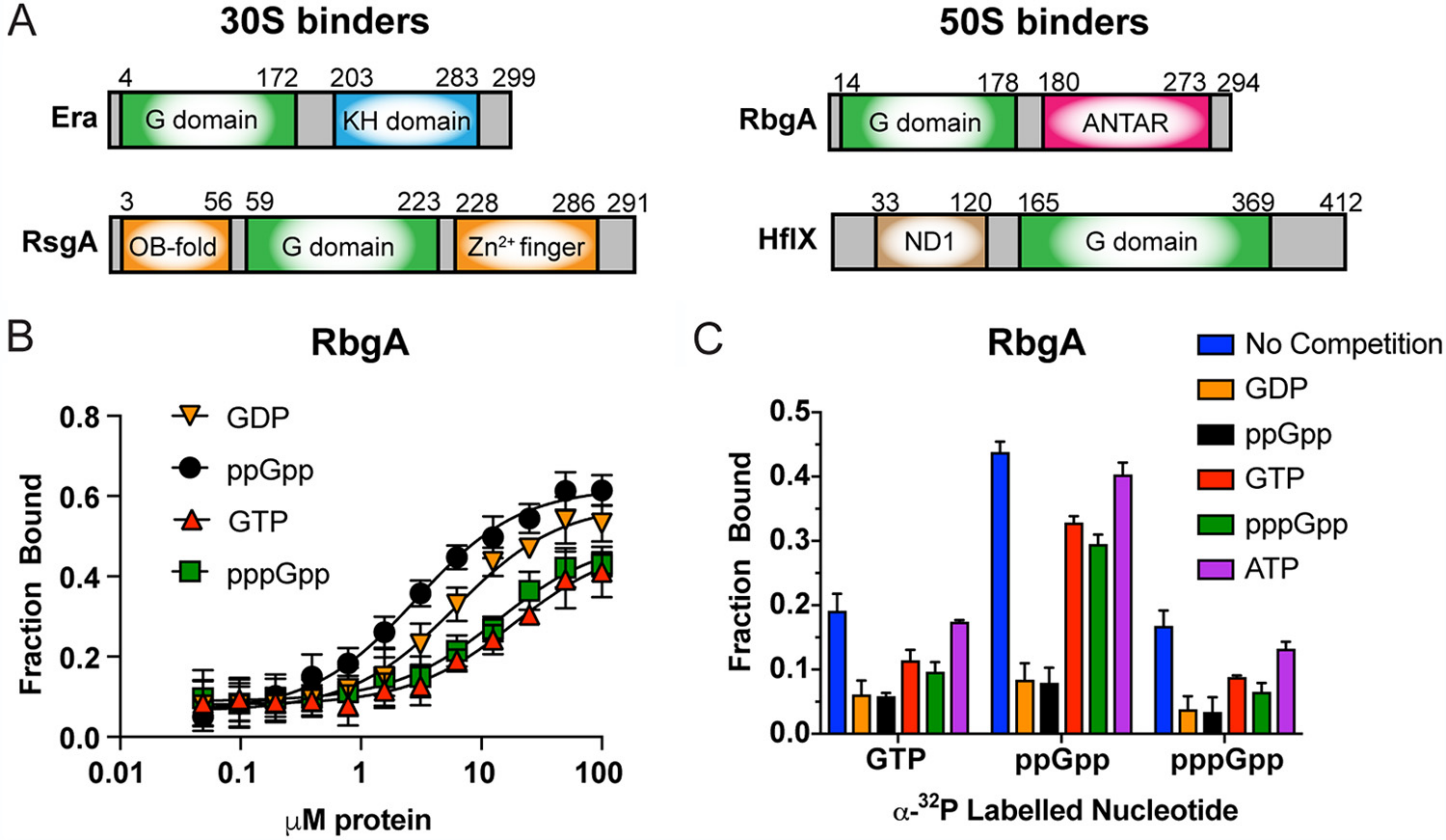
Nucleotide binding characteristics of RA-GTPases. (A) Schematic representation of the domain structure of Era, RsgA, RbgA, and HflX from S. aureus. The conserved GTPase domain (G domain) is colored in green, and accessory domains are shown. (B) Determination of binding affinities and *K_d_* values for ^32^P-labeled GTP, GDP, ppGpp, and pppGpp with purified recombinant 6×His-tagged RbgA using DRaCALA, as previously described (36). Each point is the mean average of at least three technical replicates, and error bars indicate standard deviations. (C) Binding assay (DRaCALA) of recombinant RbgA binding to ^32^P-labeled GTP, ppGpp, and pppGpp in the presence of an excess of cold competitor (GTP, GDP, ppGpp, pppGpp, or ATP). All experiments were carried out in triplicate, with error bars representing standard deviations.

The prokaryotic 70S ribosome is an essential and complex macromolecular assembly responsible for the translation of mRNA into functional proteins. It comprises a large 50S and a small 30S subunit, which consist of 33 ribosomal proteins (r-proteins L1 to L36) associated with two ribosomal RNAs (rRNA), and 21 r-proteins (S1 to S21) with one rRNA, respectively. Due to the energetic cost of ribosome synthesis and the intricacy of assembly, cofactors play a vital role in ensuring the correct conformation of the complete 70S (1). One class of assembly cofactors are the ribosome-associated GTPases (RA-GTPases), a subset of P-loop GTPases within the TRAnslation FACtor associated (TRAFAC) family, of which the proteins RsgA, RbgA, Era, and HflX are members. RA-GTPases have a highly conserved G-domain housing the catalytic G1-G5 motifs (see Fig. S1), flanked by one or more highly variable accessory domains that convey targeting and additional functionality to the enzymes (Fig. 1A) (2–6). The high degree of sequence identity (see Fig. S1A) and structural conservation (see Fig. S1B to E) between functional motifs within the nucleotide-binding pocket suggests a common mechanism of guanosine nucleotide binding among these P-loop RA-GTPases.

10.1128/mBio.02679-21.3 FIG S1 Comparison of the GTPase domains of Era, HflX, RsgA, and RbgA. (A) Domain structure and sequence alignments of the GTPase domains of S. aureus Era and HflX (top) and RsgA and RbgA (bottom). The conserved functional motifs G1 to G4 are highlighted as follows: G1, red; G2, green; G3, blue; and G4, yellow. The G5 motifs (brown) are not shown in the alignment due to low sequence conservation. Note the circular permutation of the RsgA and RbgA GTPase domains results in the G4 and G5 motifs being located N-terminal of the G1, 2 and 3 motifs. Sequence alignments were generated using Clustal Omega (80). (B to E) Structures of the GTPase domains of Aquifex aeolicus Era (adapted from PDB 3R9W) (B), E. coli HflX (adapted from PDB 5ADY) (C), B. subtilis YloQ (an RsgA homologue, adapted from PDB 5NO3) (D), and B. subtilis YlqF (an RbgA homologue, adapted from PDB 1PUJ) (E) in the GMPPNP-bound states. Functional motifs G1 (red), G2 (green), G3 (blue), and G4 (yellow) are highlighted. The bound GMPPNP ligand is colored by atom, and the bound Mg^2+^ ion is shown in magenta. The switch I (G2) region of RbgA was unresolved in the structure; therefore, the rough expected location of switch 1 is marked by a green dashed line. Download FIG S1, TIF file, 2.9 MB. Copyright © 2021 Bennison et al. 2021 Bennison et al. https://creativecommons.org/licenses/by/4.0/ This content is distributed under the terms of the Creative Commons Attribution 4.0 International license.

Due to the variation in accessory domains, each RA-GTPase associates with a distinct area of the ribosome to coordinate a maturation event. Cycling between the GTP-bound ON and GDP-bound OFF states enables these proteins to act as molecular checkpoints of ribosome assembly by monitoring the maturation state of individual subunits (7). Although it is unclear what the precise roles of RA-GTPases are in ribosomal maturation, they have been suggested to sterically prevent the premature association of other r-proteins (8). Unknown maturation events then act as activators of GTPase activity, enabling entry into the GDP-bound OFF state and subsequent dissociation from the ribosome (7). In addition to regulating the recruitment of r-proteins, RA-GTPases have been postulated to recruit RNA processing enzymes directly. For instance, the RA-GTPase Era can interact with several proteins involved in 16S rRNA maturation, including YbeY, an endonuclease involved in 16S processing in Escherichia coli (9), and CshA, a DEAD-box RNA helicase (10), pointing to a role for this group of enzymes as hub proteins that facilitate maturation events. In addition to interacting with immature subunits, these GTPases can similarly bind to mature 30S and 50S subunits in isolation, as well as while part of the 70S complex, with the latter promoting subunit dissociation *in vitro* when the RA-GTPase is in excess (4, 11, 12). While the *in situ* function of this is unclear, it may be related to the rescue of nonfunctional or incorrectly structured intermediates during stress, a function already assigned to HflX (13).

During periods of starvation, bacteria overproduce the alarmones guanosine penta- and tetraphosphate (collectively referred to as (p)ppGpp), which function as the mediators of a stress signaling system termed the stringent response (14, 15). During balanced growth, (p)ppGpp is present at slightly fluctuating basal levels and serves to maintain cellular component homeostasis and stability (16). Amid the stringent response, the concentration of (p)ppGpp within the cell rises to reach between 1 and 2 mM with a concurrent drop in GTP levels (17, 18). This results in a plethora of downstream effects during what is thought to be a highly prioritized process (19), including alterations to (i) transcription through derepression of the CodY regulon (20); (ii) translation through the binding and inhibition of several translation factors, including elongation factor G (EF-G), elongation factor Tu (EF-Tu), and bacterial initiation factor 2 (IF2) (21–23); and (iii) DNA replication, as well as regulating late-stage growth phases such as sporulation or biofilm formation (24–26). Our previous work identified the four RA-GTPases (RsgA, RbgA, Era, and HflX: Fig. 1A) in the pathogenic bacterium Staphylococcus aureus as enzymes that can bind to and are inhibited by (p)ppGpp, resulting in a negative impact on 70S ribosome assembly and growth (27).

RsgA is a nonessential, highly conserved late-stage 30S assembly cofactor (27, 28) that has been implicated in the docking of helix 44 (h44) of the 16S rRNA into the correct conformation and therefore correct maturation of the decoding center prior to subunit joining (4, 29, 30). Era is a highly conserved protein that interacts with the anti-Shine-Dalgarno sequence toward the 3′ ends of 16S rRNA and pre-16S rRNA (3) in order to monitor the RNase processing state of this region. Furthermore, since Era docking occurs at the same site as r-protein S1 adjacent to the anti-Shine-Dalgarno sequence, it can also sterically occlude initiation factor 3 (IF3) binding and hence prevent formation of the 30S preinitiation complex (pre-IC) (31). RbgA is a late-stage 50S binding protein, implicated in RNA binding and remodeling (6, 32). Finally, HflX is a 30S, 50S, and 70S binding protein that has been implicated in the splitting and subsequent repair of heat-stalled 70S ribosomes (33). HflX also contributes directly to 70S levels through GTPase-dependent splitting of the 100S hibernation complex to enable rapid recovery of active 70S ribosomes when cellular energy levels rise, a process that is inhibited when bound to (p)ppGpp (13).

The binding of pppGpp to RbgA has previously been suggested to enhance the affinity of this protein for the mature 50S subunit compared to the GTP-bound form (34). More recently, the crystal structure of S. aureus RbgA in complex with both ppGpp and pppGpp was solved, revealing a competitive mode of inhibition at the catalytic center (6). These findings have led to a proposed model wherein RbgA-(p)ppGpp likely sequesters 50S ribosomal subunits to prevent the formation of active 70S ribosomes (6). Here, we further characterize the four RA-GTPases RsgA, RbgA, Era, and HflX to investigate the relationship between RA-GTPases and stringent response-mediated control of ribosome assembly in S. aureus. We find that the 5′ diphosphate nucleotides GDP and ppGpp can bind to these enzymes with higher affinity than the 5′ triphosphate-containing GTP or pppGpp, suggesting that occupancy of the binding site is strongly dependent on a cellular excess of GTP over GDP, which occurs in proliferating and nonstressed cells (35). In contrast to previous models (6, 34), we demonstrate here that interactions with (p)ppGpp destabilize the association of RA-GTPases to the ribosome, both *in vitro* and in S. aureus. To examine mechanistically how (p)ppGpp impacts RA-GTPase-ribosome interactions, we use X-ray crystallography, revealing that (p)ppGpp binding causes the RA-GTPases to adopt a conformation similar to the inactive GDP-bound OFF state, with the switch I/G2 loop required for GTP hydrolysis extended away from the catalytic site, where it could sterically hinder interactions with the ribosome. Altogether, we propose a mechanism behind (p)ppGpp-controlled inhibition of ribosome assembly and increase our understanding of stringent response-mediated translational control by means of RA-GTPase inhibition.

## RESULTS

### RA-GTPases preferentially bind 5′ diphosphate-containing nucleotides GDP and ppGpp.

The RA-GTPases RsgA, Era, RbgA, and HflX can bind to the guanosine nucleotides GTP, GDP, ppGpp, and pppGpp. Our previous work observed higher binding affinities for ppGpp over GTP, pointing toward a difference in binding between 5′ di- or triphosphate nucleotides (27). However, these experiments did not assess the affinity of these proteins to GDP, which represents a major step in the GTPase ON/OFF cycle. Furthermore, these previous experiments were performed using recombinant proteins fused to large MBP tags, which could impact binding affinity determination. To examine the nucleotide binding affinities of these RA-GTPases for GDP in comparison to ppGpp, pppGpp, and GTP, and in the absence of a large tag, we used a differential radial capillary action of ligand assay (DRaCALA) with recombinant RsgA, RbgA, Era, and HflX fused to a smaller 6× His tag (Fig. 1B; see also Fig. S2A to C in the supplemental material) (36). In each case, the affinities of 5′ diphosphate-containing GDP and ppGpp were similar in the low μM range and were 2- to 6-fold higher than the affinities of either GTP or pppGpp (Table 1). This supports the previous observation that ppGpp is a more potent inhibitor of GTPase activity than pppGpp (27) and also provides a more accurate representation of binding affinity.

**TABLE 1:** Binding affinities

Compound	Mean binding affinity ± SEM^*a*^							
	Era		RbgA		RsgA		HflX	
	*K_d_* (μM)	B_max_	*K_d_* (μM)	B_max_	*K_d_* (μM)	B_max_	*K_d_* (μM)	B_max_
GDP	4.9 ± 0.7	0.5 ± 0.0	6.1 ± 1.1	0.5 ± 0.0	1.8 ± 0.2	0.9 ± 0.0	4.9 ± 0.7	0.7 ± 0.0
ppGpp	4.2 ± 0.6	0.4 ± 0.0	2.9 ± 0.4	0.6 ± 0.0	2.2 ± 0.2	0.9 ± 0.0	3.4 ± 0.4	0.6 ± 0.0
GTP	11.5 ± 1.6	0.3 ± 0.0	18.5 ± 5.4	0.4 ± 0.0	3.6 ± 0.4	0.8 ± 0.0	ND	0.7 ± 0.3
pppGpp	13.9 ± 4.7	0.2 ± 0.0	13.8 ± 4.0	0.4 ± 0.0	10.1 ± 2.2	0.4 ± 0.0	ND	0.5 ± 0.2

a B_max_ values indicate the fraction bound.

10.1128/mBio.02679-21.4 FIG S2 Examination of the binding of GDP, ppGpp, GTP, and pppGpp to RA-GTPases by DRaCALA. (A to C) Determination of binding affinities and *K_d_* values for ^32^P-labeled nucleotides to purified recombinant 6×His-tagged RsgA (A), Era (B), and HflX (C). *K_d_* values were determined from the binding curves as previously described (36). (D to F) DRaCALA binding assay of recombinant RsgA (D), Era (E), and HflX (F) binding to ^32^P-labeled GTP, ppGpp, and pppGpp in the presence or absence of 100 μM cold competitors (GTP, GDP, ppGpp, pppGpp, and ATP). All experiments were carried out in triplicate, with error bars representing standard deviations. Download FIG S2, TIF file, 0.9 MB. Copyright © 2021 Bennison et al. 2021 Bennison et al. https://creativecommons.org/licenses/by/4.0/ This content is distributed under the terms of the Creative Commons Attribution 4.0 International license.

Structural data places (p)ppGpp within the GTP-binding site of the RA-GTPase RbgA (6), indicating a competitive mode of inhibition. To examine whether this inhibition is consistent across the four RA-GTPases, competition assays were performed in which the binding of a radiolabeled nucleotide was challenged with an excess of unlabeled nucleotides (Fig. 1C; see also Fig. S2D to F). Based on our measured affinities (Table 1), we speculate that both GDP and ppGpp will outcompete other nucleotides for occupancy of the binding site. In each case, the addition of cold unlabeled nucleotide reduced the occupancy of the labeled nucleotide, with the exception of the ATP control. This is likely due to the much lower affinity of RA-GTPases for adenosine bases conveyed by a contact from the conserved aspartate residue of the G4 motif to the 2-amino group of the guanosine base (37). A hierarchy of binding could be established depending on the level of competition provided by each unlabeled nucleotide, with GDP and ppGpp competing more effectively (Fig. 1C; see also Fig. S2D to F). Although these data are from *in vitro* experiments, they suggest that the GTP occupancy, and hence the activity, of these RA-GTPases in the cell could be strongly dependent on the excess of GTP over GDP and ppGpp that occurs during exponential growth when ribosomal biogenesis is at its peak (17, 35). This ratio changes during stationary phase and upon induction of the stringent response, when cellular GTP levels decrease with a concurrent rise in (p)ppGpp (17, 38), which could shift binding to favor a ppGpp-bound state. The greater affinity of these RA-GTPases to diphosphate-containing nucleotides could hence aid a rapid transition between the GTP-bound and ppGpp-bound states under conditions of stress.

### Interactions with (p)ppGpp reduce the affinity of RA-GTPases for the ribosome.

**FIG 2:**
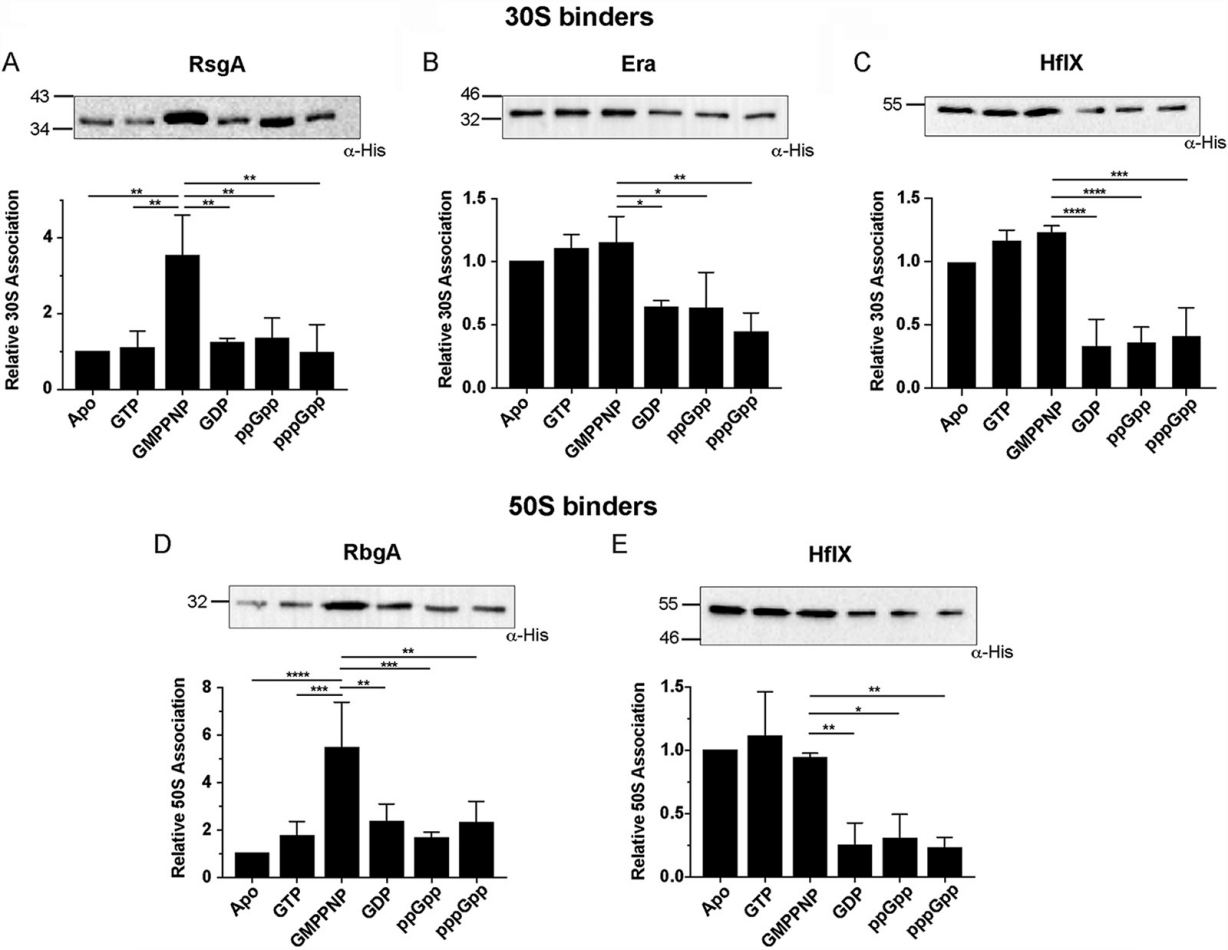
(p)ppGpp and GDP binding reduces RA-GTPase association to the ribosome. (A to C) purified 70S ribosomes were preincubated with His-tagged RsgA (A), Era (B), and HflX (C) in the absence or presence of GTP, GMPPNP, GDP, ppGpp, or pppGpp. After subunit separation and precipitation, bound proteins were detected in the 30S fraction using HRP-conjugated α-His antibodies. (D and E) ribosomes were incubated with RbgA (D) and HflX (E) in the absence or presence of GTP, GMPPNP, GDP, ppGpp, or pppGpp. Bound proteins were detected in the 50S fraction using HRP-conjugated α-His antibodies. Experiments were carried out in triplicate or quadruplicate, and one representative image (top) from each group is shown. The signal intensities relative to the apo state of all repeats are plotted (bottom), with error bars representing standard deviations. Statistical analysis was performed using a one-way ANOVA, followed by Tukey’s multiple-comparison test (*, *P < *0.05; **, *P < *0.01; ***, *P < *0.001; ****, *P < *0.0001).

It is well characterized that rRNA transcription decreases during the stringent response (39). In addition, the GTPase activity of ribosome assembly cofactors is inhibited by (p)ppGpp, both of which contribute to a reduction in mature ribosomes within the cell (27). To examine mechanistically how (p)ppGpp-GTPase interactions affect the ability of RA-GTPases to associate with ribosomal subunits, we examined the association of each GTPase to either the 30S or 50S ribosomal subunit in the presence of GDP, GTP, ppGpp, and pppGpp, as well as GMPPNP, a nonhydrolyzable analogue of GTP. The production and isolation of immature subunits, which can comprise several different immature states, introduces a large degree of heterogeneity, and so here we, and others, use homogenous mature particles as a system to examine the binding event, rather than the role of GTPase activity in downstream maturation (11, 12, 29, 40, 41). His-tagged GTPases were preincubated with highly pure, salt-washed 70S S. aureus ribosomes in a low-magnesium buffer to encourage ribosomal subunit dissociation, and the amount of each GTPase associated with each of the subunits was quantified by Western immunoblotting with anti-His antibodies after sucrose gradient separation. Binding to the 30S was observed for RsgA, Era, and HflX, while both RbgA and HflX bound to the 50S (Fig. 2). Unlike the other RA-GTPases, HflX was able to associate to both the 30S and 50S ribosomal subunits (Fig. 2C and E) in line with previous observations (42). In all cases, we observed a marked decrease in association of each GTPase to the 30S or 50S subunits in the presence of GDP, ppGpp, and pppGpp compared to the GMPPNP-bound state (Fig. 2). For Era and HflX, there was a similar level of subunit association when in the apo, GTP, or GMPPNP-bound states, compared to a 2-fold reduction in ribosome binding when incubated with GDP, ppGpp, or pppGpp (Fig. 2B, C, and E), suggesting that these GTPases can associate with the ribosome in the unbound state. The ability of Era to bind the 30S in the absence of nucleotides has been reported previously, where it has been suggested that the apo form can bind to mature 30S subunits in a distinct conformation to either the GDP- or GTP-bound states (3, 12). The patterns exhibited by RsgA and RbgA were slightly different, with strong binding in the GMPPNP-bound state, whereas 3- to 6-fold weaker binding was observed in the apo-, GTP-, GDP-, ppGpp-, and pppGpp-bound states (Fig. 2A and B). It is worth noting that previous studies have suggested that the association of RbgA with the 50S subunit is enhanced in the presence of pppGpp (34), a finding that is not replicated here. The apparent effect of ppGpp and pppGpp on ribosome association was comparable, which is not reflective of the differences in affinity (Fig. 1B; see also Fig. S2A to C), although under the conditions tested here the excess of nucleotide would maintain an equilibrium favoring the nucleotide-bound state. Furthermore, the four RA-GTPases were found to be unable to hydrolyze pppGpp, and as such conversion of pppGpp to ppGpp was not responsible for the similar degree of inhibition of association. We postulate that the low level of binding observed when preincubated with GTP is due to GTP hydrolysis during the 16-h centrifugation step, likely causing the GTPases to enter the GDP-bound state and dissociate. This, in turn, may be enhanced by the higher affinity of GDP for these GTPases compared to GTP (Table 1). From these data, we show that association of RsgA and RbgA to ribosomal subunits is favored while in the GTP-bound state and that the interaction of all four RA-GTPases with the ribosome is reduced when in the GDP-, ppGpp-, or pppGpp-bound states.

### Binding kinetics of RA-GTPase-ribosome interactions.

**FIG 3:**
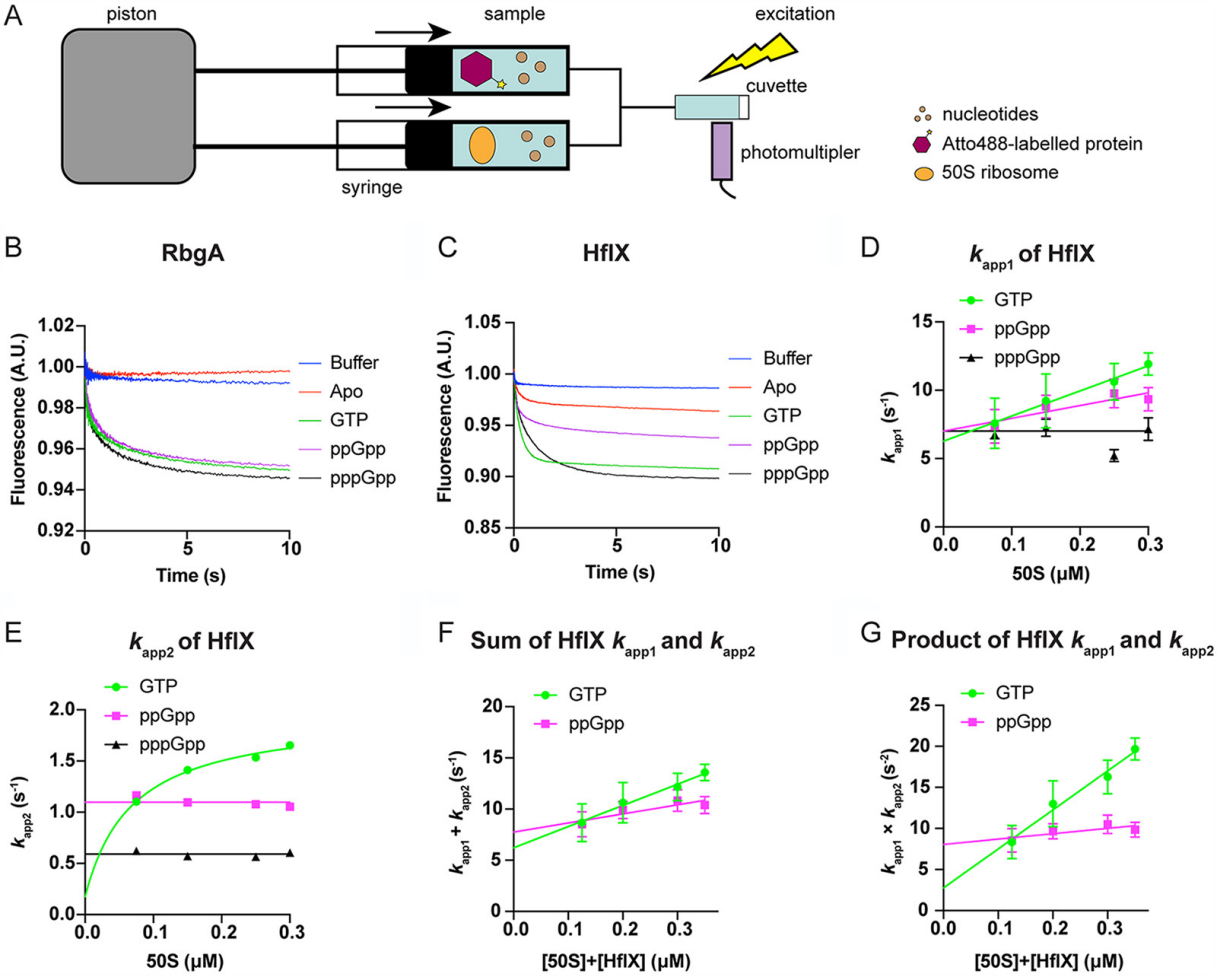
Stopped-flow kinetic parameters of RA-GTPase association to the ribosomal subunits. (A) Schematic representation of the experimental setup for stopped-flow analysis. Nucleotides (brown circles), 50S subunits (orange oval), and Atto488-labeled RA-GTPases (purple hexagon) are indicated. Arrows indicate the direction of syringe movement. Atto-488 was excited using a 470-nm LED, and fluorescence was detected through a 515-nm long-pass filter. (B and C) Fluorescent change upon mixing 0.2 μM RbgA-Atto488 (B) or HflX-Atto488 (C) with 0.2 μM 50S ribosomal subunits in the presence of 100 μM GTP, ppGpp, and pppGpp or in the apo state using the stopped-flow fluorescence apparatus. Fluorescently labeled protein was also mixed with buffer lacking 50S subunits as a mixing control. Fluorescence of the reaction was tracked using exponential sampling for 10 s, and each curve represents the mean average of at least five technical replicates. (D) *k*_app1_ dependence on 50S concentration for HflX complexed with GTP (green), ppGpp (pink), and pppGpp (black). (E) Same as for panel D for the *k*_app2_ dependence. (F and G) Sum and product analyses of apparent rates during HflX association to the 50S subunit. HflX-Atto488 (0.05 μM) was mixed with increasing titrations of 50S ribosomal subunits over the fluorescently labeled protein in the presence of 20 μM GTP or ppGpp. The resultant traces (see Fig. S4) were analyzed by nonlinear regression using two exponential terms. The sum (F) and product (G) of apparent rates (*k*_app1_ [D] and *k*_app2_ [E]) were plotted as a function of the total concentration of the 50S subunits and HflX protein to determine the microscopic constants *k*_1_, *k*_-1_, *k*_2_, and *k*_-2_ (Table 2) and the resulting dissociation constant (*K_d_*) (see Materials and Methods). Error bars represent the standard deviations of the apparent rates of four or more individual traces (D and E) or the standard errors of the two-step analysis (F and G).

To gain further insight into the binding mechanism and how (p)ppGpp reduces the association of RA-GTPases to the ribosomal subunits, we used a stopped-flow technique with fluorescent derivatives of the RA-GTPases (Fig. 3A). Structural predictions of all four RA-GTPases were built by homology modeling using available structures to assess the availability of suitable residues for fluorescence labeling (see Fig. S3A and B) (43). Both RbgA and HflX were amenable to covalent linkage to the fluorophore Atto-488 using maleimide chemistry with exposed cysteine residues. RbgA contains one wild-type cysteine residue (C277) that is surface exposed in the B. subtilis crystal structure (PDB 1PUJ) and is located toward the C terminus of the protein (see Fig. S3A). Based on the E. coli structure (PDB 5ADY), HflX contains two cysteines (see Fig. S3B). C330 is predicted to be surface exposed and therefore amenable to labeling, whereas C45 is buried and is expected to show low accessibility for fluorescent labeling. Era, on the other hand, lacks any cysteine residues, while RsgA contains three conserved cysteine residues that coordinate the Zn^2+^ ion within the Zn^2+^-finger domain (ZNF), and as such both Era and RsgA were not suitable for labeling. Both Atto488-labeled RbgA and HflX retained wild-type levels of GTPase activity, which can still be inhibited by ppGpp (see Fig. S3C and D).

10.1128/mBio.02679-21.5 FIG S3 Labeling sites and GTPase activity of Atto488-labelled proteins. (A and B) Predicted full-length structures of RbgA (A) and HflX (B). Cysteine residues are shown in red, and protein domains are indicated. Cysteine residues amenable for labeling with Atto488-maleimide are shown as spheres. Structures were predicted through homology modeling (SWISS MODEL server) (43), using template PDBs 1PUJ (chain A) and 5ADY, respectively. (C and D) GTPase activity of 0.1 μM wild-type and Atto488-labeled RbgA (C) and HflX (D) in the presence or absence of 100 μM ppGpp. Reaction mixtures containing 1 μM GTP spiked with ^32^P-labeled GTP, 0.1 μM RA-GTPase, and 0.1 μM 70S ribosomes to stimulate GTPase activity were incubated at 37°C for 60 min. Control reactions lack the RA-GTPases. Enzymatic activity was monitored by TLC and quantified by using ImageQuant. Experiments were performed in triplicate, and error bars represent standard deviations. Download FIG S3, TIF file, 1.0 MB. Copyright © 2021 Bennison et al. 2021 Bennison et al. https://creativecommons.org/licenses/by/4.0/ This content is distributed under the terms of the Creative Commons Attribution 4.0 International license.

10.1128/mBio.02679-21.6 FIG S4 Stopped-flow kinetic parameters of RA-GTPases associating to the 50S ribosome. (A to C) 0.075 μM Atto488-labeled RbgA was rapidly mixed with increasing molar excesses of 50S ribosomal subunits in the presence of GTP (A), ppGpp (B), or pppGpp (C). (D to F) 0.05 μM HflX-Atto488 was mixed with increasing molar excesses of 50S in the presence of GTP (D), ppGpp (E), and pppGpp (F). Sampling was carried out in an exponential manner over a 10-s period, and resulting traces were analyzed by nonlinear regression using two exponential terms, shown as a solid black line. Each trace is the average of at least five replicates. (G to J) 0.075 μM RbgA-Atto488 was mixed with increasing titrations of 50S ribosomal subunits at molar excess over fluorescently labeled protein in the presence of 20 μM GTP, ppGpp, or pppGpp. The resultant traces (A to C) were analyzed by nonlinear regression using two exponential terms. The sum (I) and product (J) of apparent rates (*k*_app1_ [G] and *k*_app2_ [H)) were plotted as a function of the total concentration of the 50S subunit and RbgA protein, and the dissociation constant (*K_d_*) was calculated as specified in Materials and Methods. Error bars represent the standard deviations of the apparent rates of four or more individual traces (G and H) or the standard errors of the two-step analysis (I and J). Download FIG S4, TIF file, 1.1 MB. Copyright © 2021 Bennison et al. 2021 Bennison et al. https://creativecommons.org/licenses/by/4.0/ This content is distributed under the terms of the Creative Commons Attribution 4.0 International license.

Using the fluorescent variants of RbgA and HflX, we studied the binding mechanism of both to the 50S ribosomal subunit in the GTP-, ppGpp-, and pppGpp-bound states. First, the fluorescence change of each labeled protein was measured upon the interaction with activated mature ribosomal subunits in the presence of different nucleotides (Fig. 3B and C). RbgA showed no change in fluorescence while in the apo state, indicating a lack of interaction with the ribosome. On the other hand, all nucleotide-bound states showed a large decrease in fluorescence when mixed with the 50S subunit, consistent with some level of 50S association taking place when bound to GTP, ppGpp, or pppGpp (Fig. 3B). HflX, on the other hand, exhibited a fluorescence change upon mixing with the 50S subunit in the absence or presence of all tested nucleotides (GTP, ppGpp, and pppGpp), which could be taken as a direct measure of ribosome association changing the chemical environment of the fluorophore (Fig. 3C).

Next, we used a constant concentration of protein in the presence of 200-fold excess of each nucleotide and titrated it with increasing concentrations of ribosomal subunits (see Fig. S4). Time traces appeared biphasic for both RA-GTPases independent of the nucleotide bound. Analysis of the fluorescent time traces with a double exponential equation yielded the apparent rates of association (*k*_app1_ and *k*_app2_) (Fig. 3D and E; see also Fig. S4G and H), in accordance with a binding mechanism composed of two sequential steps. Thus, the mechanism describing the following interaction:

$$P+S\begin{array}{c} k_{1}\\ \;⇌\\ k_{-1} \end{array}\;PS\text{'}\begin{array}{c} k_{2}\\ ⇌\\ k_{-2} \end{array}PS$$

consists of an initial interaction and subsequent stabilization of the factor on the ribosome, where *P* is the protein, *S* is the ribosomal subunit, *PS′* is the transient complex, and *PS* is the stable complex.

For two-step reactions, the apparent rate under conditions tested, *k*_app1_, is expected to increase linearly with increasing ligand concentration. On the other hand, *k*_app2_ is expected to align to a hyperbolic relationship as ligand concentration increases (44). This was the case for HflX complexed with GTP (Fig. 3D and E). Thus, productive binding of the RA-GTPase appears to occur through two steps. When HflX was incubated with ppGpp, the *k*_app1_ increased linearly (Fig. 3D), while *k*_app2_ did not depend on ribosome concentration (Fig. 3E), indicating that ppGpp hampers the accommodation step of the binding mechanism. On the other hand, if HflX was complexed with pppGpp, neither *k*_app_ value depended on 50S concentration, indicating that the alarmone drastically affects the mechanism of HflX binding. In this case, the reaction appears to be rate limited by an isomerization step of the RA-GTPase at 5 s^−1^ (Fig. 3D). The linear increase in *k*_app1_ was 2-fold greater for GTP than for ppGpp or pppGpp (Fig. 3D), suggesting a greater rate of the fast-phase reaction. The *k*_app2_ of the GTP-bound form showed a hyperbolic relationship tending to 2 s^−1^, while the linear relationship when bound to ppGpp was steady at 1.0 s^−1^ (Fig. 3E). This suggests that the second, slow-phase reaction is taking place while HflX is bound to GTP but is reduced 4-fold when bound to ppGpp. In addition, this suggests that one or more of the microscopic constants which contribute to the *k*_app2_ in the two-step association reaction remains incomplete while in the ppGpp-bound state.

Next, we used the sum and product of the *k*_app1_ and *k*_app2_ of each reaction (Fig. 3F and G) to estimate approximate microscopic constants defining the reaction for the GTP- and ppGpp-bound HflX (Table 2). ppGpp reduced the value of the initial binding constant *k*_1_, while drastically affecting *k*_2_, indicating that the alarmone hampers proper accommodation of HflX on the subunit (Fig. 3F and G and Table 2). On the contrary, the dissociation rate constants *k*_-1_ and *k*_-2_ appeared less affected by ppGpp, remaining similar to those observed during the GTP-bound state (Table 2). Altogether, our data indicate that (p)ppGpp induces a nonproductive conformation of HflX, reducing the binding progression with the ribosomal subunit.

**TABLE 2:** Association (*k*_1_ and *k*_2_) and dissociation (*k*_–1_ and *k*_–2_) rate constants and approximate dissociation constant (*K_d_*) of HflX and RbgA binding to 50S ribosomes in various nucleotide-bound states

GTPase	Nucleotide	Mean ± SEM^*a*^				
		*k*_1_ (s^−1^)	*k*_–1_ (s^−1^)	*k*_2_ (s^−1^)	*k*_–2_ (s^−1^)	*K_d_* (μM)
HflX	GTP	20.7 ± 1.7	3.9 ± 0.5	1.6 ± 0.4	0.7 ± 0.3	0.06 ± 0.03
	ppGpp	8.9 ± 3.3	7.0 ± 1.0	∼0 ± 0.5	1.1 ± 0.2	1.2 ± 0.9
RbgA	GTP	13.3 ± 0.9	1.3 ± 0.4	0.14 ± 0.7	0.6 ± 0.7	0.08 ± 0.08
	ppGpp	15.7 ± 6.7	3.6 ± 3.4	∼0 ± 1.3	0.6 ± 1.2	0.3 ± 0.6
	pppGpp	12.6 ± 2.6	5.4 ± 1.3	0.4 ± 4.4	0.5 ± 4.4	0.2 ± 2.0

a Negative rate values for *k*_2_ approximated ∼0 s^−1^. HflX complexed with pppGpp did not obey a two-step model for interaction and appeared rate-limited by an isomerization step at 5 s^−1^ (Fig. 3D). Error values shown represent the standard error of the two-stage analysis.

In the case of RbgA, all three tested nucleotides adhered to a two-step mechanism model, with *k*_app1_ increasing linearly with 50S concentration, while *k*_app2_ appeared to be hyperbolic (see Fig. S4G and H). Further analysis to estimate the microscopic constants indicated that (p)ppGpp increased the dissociation rate constant *k*_-1_ compared to GTP, whereas its association velocity *k*_1_ appeared largely unaffected (Table 2; see also Fig. S4I and J). Interestingly, ppGpp also reduced the accommodation rate constant *k*_2_, although less drastically than HflX, whereas pppGpp did not. Altogether, our results indicate that (p)ppGpp can program RbgA to adopt different conformations that ultimately reduce their binding affinity for the ribosome (Table 2), although to a lesser extent than for HflX.

For both RA-GTPases, it appears that the main difference on a kinetic level, in agreement with our previous observations regarding the accommodation step, is that the binding of (p)ppGpp affects the initiation of the slow-phase reaction (*k*_2_). (p)ppGpp would therefore prevent the stable association of the RA-GTPase with the ribosomal subunit. The affinity is further affected by the increased *k*_–1_ in the alarmone-bound states, which, in addition to the lack of the second phase reaction while bound to (p)ppGpp, may lead to an increase in reversal reactions, enhancing the dissociation of the RA-GTPase from the ribosomal subunits. Altogether, the kinetic data are in accordance with the observation by Western immunoblotting (Fig. 2) that these RA-GTPases associate less readily in the presence of the stringent response alarmones (p)ppGpp. Specifically, (p)ppGpp appear to affect the forward reactions, consistent with inducing a nonproductive conformation of the RA-GTPases. This could lead to diminished association of RA-GTPases to ribosomes at physiologically relevant alarmone ratios during the stringent response, when (p)ppGpp becomes the dominant cytosolic guanine nucleotide in at least a 2-fold excess over GTP (17), thus impairing ribosome maturation under stress.

### Association of the RA-GTPase Era to the 30S subunit decreases upon induction of the stringent response.

**FIG 4:**
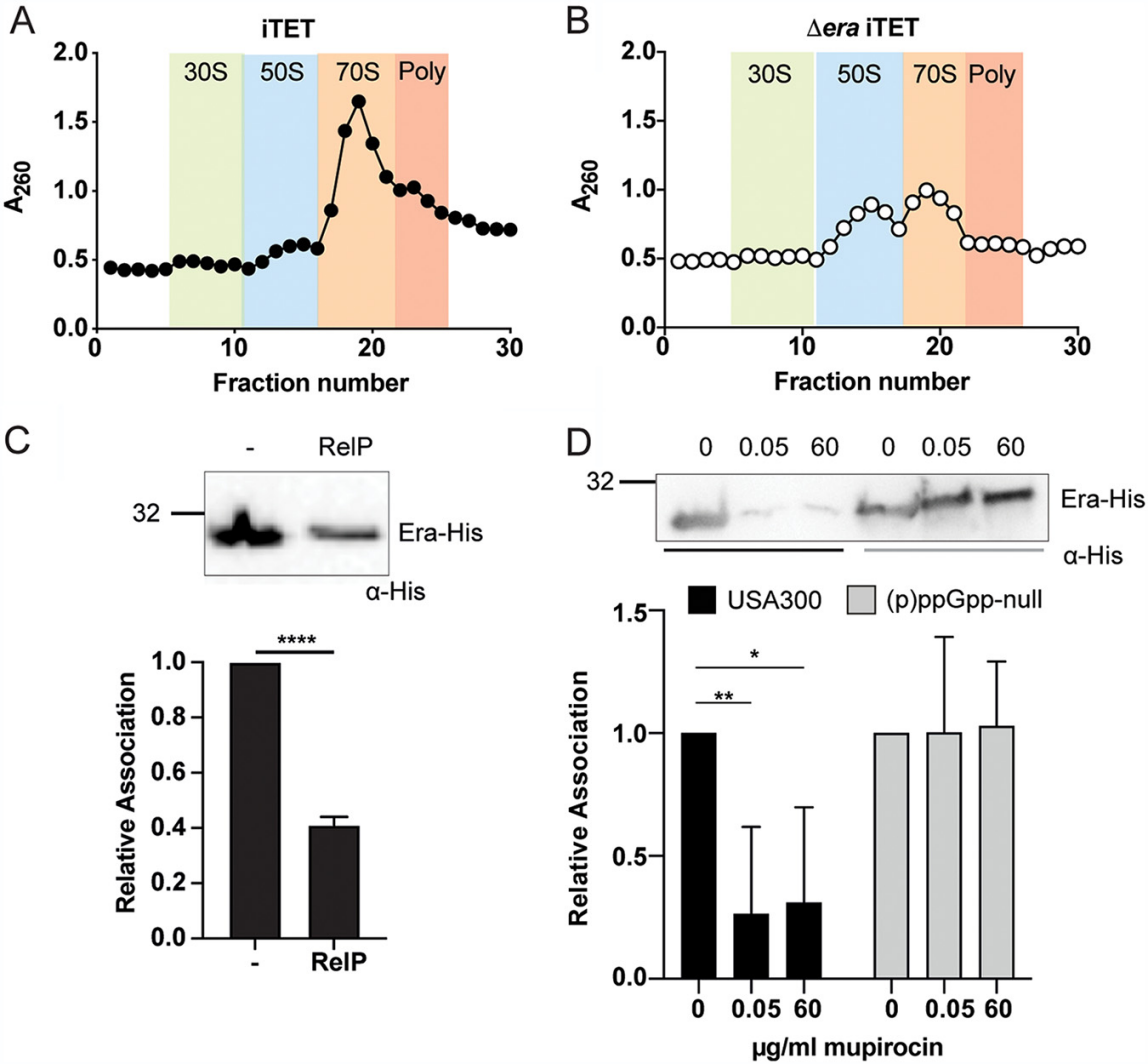
Association of Era to the 30S subunit is reduced under stringent conditions. (A and B) Ribosome profiles of the S. aureus USA300 iTET (A) and USA300 Δ*era* iTET (B) strains. RNA content was analyzed at an absorbance of 260 nm. All experiments were performed in triplicate, with one representative profile included for each strain. Expected regions for 30S subunits (green), 50S subunits (blue), 70S ribosomes (orange), and polysomes (pink) are highlighted. (C) Ribosome association of Era-His from USA300 Δ*era* iTET-*era*-His pALC2073 and USA300 Δ*era* iTET-*era*-His pALC2073-*relP* strains. (Top) Western immunoblot showing the association of Era-His to 30S ribosomes. USA300 Δ*era* iTET-*era*-His pALC2073 (left) and USA300 Δ*era* iTET-*era*-His pALC2073-*relP* (right) strains were grown to an OD_600_ of 0.8 and induced with 100 ng/ml Atet for 30 min to induce the expression of both Era-His and RelP. Ribosomal subunits were separated, and the amount of Era-His associated in each strain was detected using HRP-conjugated α-His antibodies. Experiments were carried out in triplicate, and one representative image is shown. (Bottom) The mean signal intensities relative to the empty vector control (USA300 Δ*era* iTET-*era*-His pALC2073) sample of all repeats were plotted, with error bars representing standard deviations. Statistical analysis was carried out using unpaired, two-tailed *t* testing (****, *P < *0.0001). (D) Ribosome association of Era-His from USA300 Δ*era* iTET-*era*-His and USA300 (p)ppGpp-null Δ*era* iTET-*era*-His strains. (Top) Western immunoblot showing the association of Era-His to 30S ribosomes. Both strains were grown to an OD_600_ of 0.6, and Era expression was induced with 100 ng/ml Atet for 30 min, followed by 0.05 or 60 μg/ml mupirocin for 15 min to induce the stringent response. Ribosomal subunits were separated, and the amount of Era-His associated was detected with HRP-conjugated α-His antibodies. Experiments were carried out in triplicate, and one representative image is shown. (Bottom) The mean signal intensities relative to the zero mupirocin sample of all repeats were plotted, with error bars representing the standard deviations. Statistical analysis was carried out using a one-way ANOVA, followed by Tukey’s multiple-comparison test (*, *P < *0.05; **, *P < *0.01).

Upon induction of the stringent response, cellular levels of (p)ppGpp increase, while concentration of GTP drops (38). Having observed decreased association of RA-GTPases to ribosomal subunits *in vitro*, we wanted to examine the interaction under more physiologically relevant conditions. To investigate RA-GTPases interactions with the ribosome in the bacterial cell, we used an *era* deletion mutant in a community-acquired methicillin-resistant S. aureus (CA-MRSA) USA300 strain that we had previously constructed (10). This strain has a growth defect (see Fig. S5A) and has an abnormal cellular ribosomal profile compared to the wild type, with an accumulation of 50S subunits and a loss of 70S ribosomes (Fig. 4A and B) (10, 45, 46), suggesting that the absence of this GTPase is preventing mature ribosome formation and growth. To establish whether induction of the stringent response in bacterial cells leads to a decrease in the association of Era to the 30S subunit, the *era* mutant was complemented with an Atet-inducible 6×His-tagged version of *era* using the iTET vector. To allow for the overexpression of (p)ppGpp, we also introduced an Atet-inducible copy of the (p)ppGpp synthetase *relP* on the compatible pALC2073 vector, yielding strain USA300 Δ*era* iTET-*era*-His pALC2073-*relP*. We then grew cells to exponential phase and induced expression of both Era-His and RelP through treatment with 100 ng/ml Atet for 30 min, inducing the stringent response via rapid enzymatic production of (p)ppGpp (47, 48). Cells were lysed and applied to 10 to 40% sucrose gradients in ribosome dissociation buffer for subunit separation via isopycnic ultracentrifugation. Normalized 30S pools were analyzed for associated Era-His using α-His Western immunoblotting (Fig. 4C). Crude lysates sampled prior to loading on the sucrose gradients were also analyzed to ensure equal loading and equal expression of Era-His between samples (see Fig. S5B). In agreement with the *in vitro* Western immunoblot data, the relative association of Era-His to the ribosome decreased at least 2.5-fold upon induction of the stringent response (Fig. 4C). This decrease was also observed after exposing an Era-His-expressing strain to mupirocin, an antibiotic that inhibits isoleucyl tRNA synthetase and is known to activate the stringent response in S. aureus (49). Here, Era-His exhibited a similar decrease in ribosome association after treatment with either 0.05 or 60 μg/ml mupirocin (Fig. 4D; see also Fig. S5C). However, this decrease is not seen in mupirocin-exposed cultures of a strain that lacks the three (p)ppGpp synthetases, and so this decrease is (p)ppGpp specific (Fig. 4D). Altogether, these *in vitro* and bacterial data support a model in which the stringent response impairs 70S ribosome assembly by disrupting the association of RA-GTPases with the immature ribosomal subunits, thus preventing correct ribosome maturation.

10.1128/mBio.02679-21.7 FIG S5 Association of Era to the 30S subunit is reduced under stringent conditions. (A) Growth curve of S. aureus strains USA300 iTET, USA300 Δ*era* iTET, and USA300 Δ*era* iTET-*era*-His. Overnight cultures were diluted to an OD_600_ of 0.05 and grown for 8 h in the presence of 100 ng/ml Atet. Experiments were carried out in triplicate, with error bars representing standard deviations. (B) Analysis of the expression levels of Era-His from the USA300 Δ*era* iTET-*era*-His pALC2073 and USA300 Δ*era* iTET-*era*-His pALC2073-*relP* strains after induction with 100 ng/ml Atet for 30 min. Crude lysates were analyzed using α-His Western immunoblotting for Era-His content. Experiments were carried out in triplicate, with one representative image shown. (C) Analysis of the expression levels of Era-His from USA300 Δ*era* iTET-*era*-His and USA300 (p)ppGpp-null Δ*era* iTET-*era*-His strains after induction with 100 ng/ml Atet for 30 min and mupirocin (0, 0.05, or 60 μg/ml) for 15 min. The amount of Era-His in crude lysates prior to loading on gradients was detected using HRP-conjugated α-His antibodies. Recombinant Era-His protein was also loaded as a control for protein size and identification (last lane). Experiments were carried out in triplicate, and one representative image is shown. Ladder sizes are indicated on the left. Download FIG S5, TIF file, 0.9 MB. Copyright © 2021 Bennison et al. 2021 Bennison et al. https://creativecommons.org/licenses/by/4.0/ This content is distributed under the terms of the Creative Commons Attribution 4.0 International license.

### Crystallization of RsgA in the apo- and ppGpp-bound states.

GTPases act as molecular switches, cycling between OFF (GDP-bound) and ON (GTP-bound) states. Structural studies of numerous GTPases have reported distinct conformations for both states, which are determined by the movement of the flexible switch I/G2 loop and the switch II/G3 loop (50). Often described as a loaded-spring mechanism, the conformational change occurs upon hydrolysis of GTP or the subsequent γ-phosphate release. Both switch I/G2 and switch II/G3 are responsible for coordinating the Mg^2+^ cofactor, which interacts with the γ-phosphate of GTP via a conserved threonine residue in G2 and a glycine in G3. Upon hydrolysis of the γ-phosphate and P_i_ dissociation, the protein relaxes into the OFF conformation.

**FIG 5:**
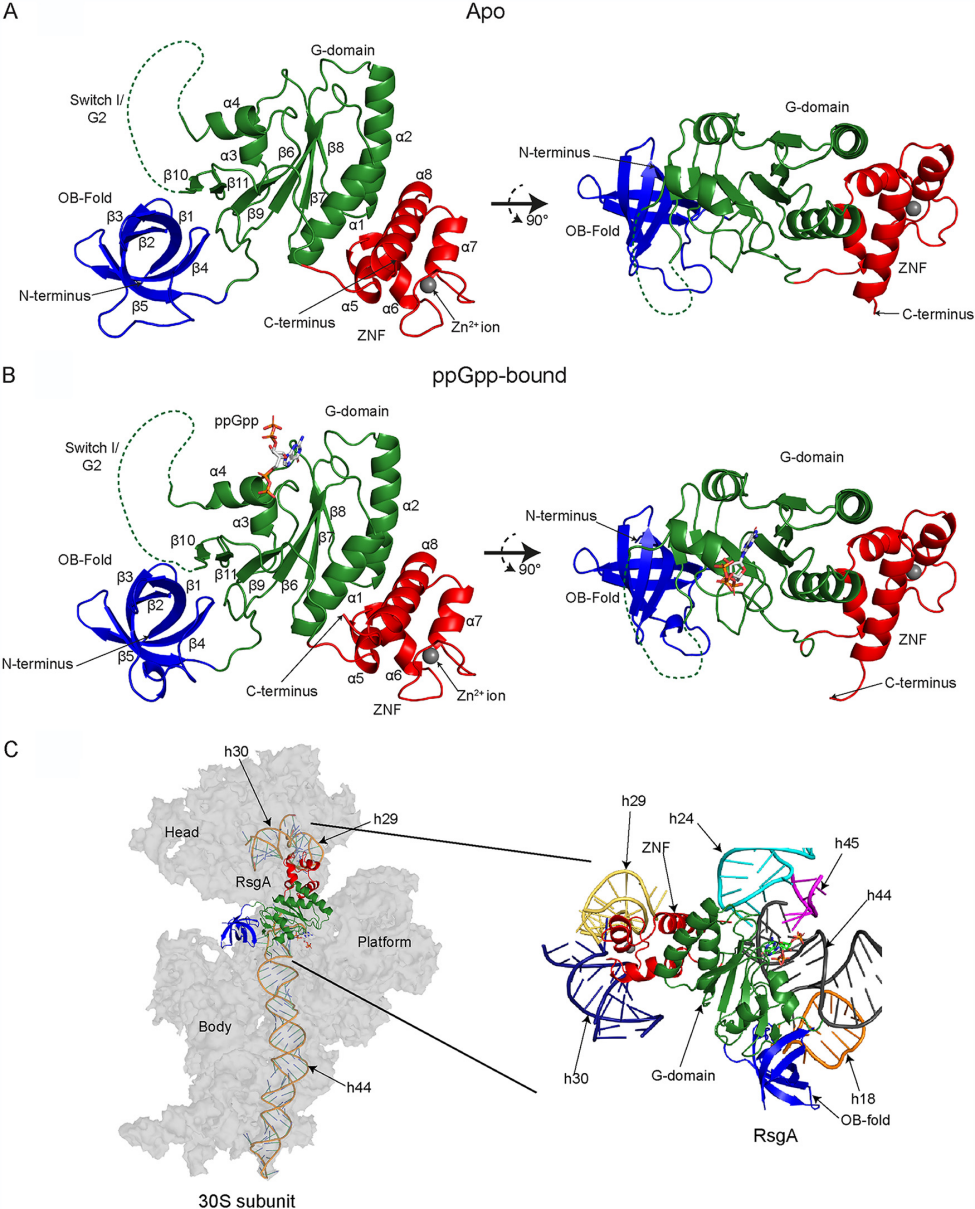
Structure of RsgA in the apo- and ppGpp-bound states. (A and B) Crystal structures of RsgA in the apo state (PDB 6ZJO) (A) and bound to ppGpp (PDB 6ZHL) (B). The structures are colored by domain, with the N-terminal OB-fold colored blue, the central G-domain colored green, and the C-terminal Zn^2+^-finger (ZNF) domain colored red. Structural details, including α-helices, β-sheets, ligands, termini, and domains, are labeled. The expected positions of the switch I/G2 loops, as determined by comparison with RsgA homologues in the GMPPNP-bound state, are indicated using a dotted line, despite the lack of electron density surrounding this feature. For both panels A and B, a 90° rotation around a horizontal axis is shown. (C) The RsgA binding site on the 30S ribosomal subunit. RsgA-ppGpp (PDB 6ZHL; this study) was overlaid onto the model of YjeQ-GMPPNP (PDB 5UZ4, chain Z [53]) using C_α_ alignment, relative to the 30S ribosomal subunit (PDB 5UZ4, chain A [53]). The RsgA model is shown as a cartoon representation, colored by domain as described above. The 30S subunit is shown in gray, with interacting rRNA helices shown as cartoon representations to highlight the RsgA recognition sites, as labeled. The bound ppGpp ligand is colored by atom: carbon, gray; nitrogen, blue; oxygen, red; and phosphorous, orange. (Inset) Cartoon representation of the rRNA helices that constitute the RsgA binding site on the 30S subunit. Target rRNA helices are colored as follows: h24, cyan; h18, orange; h29, yellow; h30, navy blue; h44, gray; and h45, magenta.

To look more at the mechanism of (p)ppGpp-mediated inhibition of RA-GTPases associating with ribosomal subunits, we solved the structures of RsgA in both the apo- (Fig. 5A) and ppGpp-bound (Fig. 5B) states by X-ray crystallography (see Table S2) in order to compare to already-available GMPPNP- and GDP-bound structures. The 1.94-Å structure of RsgA complexed with ppGpp reveals the presence of the nucleotide unambiguously represented in the electron density map (see Fig. S6A), whereas the apo structure at 2.01 Å lacks any electron density in the nucleotide binding pocket. The overall structure of RsgA consists of three domains: the N-terminal OB-fold, the central GTPase domain, and a C-terminal ZNF (Fig. 5A). Both the OB-fold and the ZNF domains are involved in nucleotide recognition (51, 52) and target RsgA to the 30S ribosomal subunit, where they contact major helices of the 16S rRNA (Fig. 5C). The OB-fold is situated between h18 and h44, with the loop connecting β_1_ and β_2_ recognizing the minor groove of h44 adjacent to the 30S acceptor site (4). The ZNF contacts the 30S head domain, making backbone contacts with h29 and h30, close to the interaction site of the P-site tRNA (4, 53). In E. coli RsgA (YjeQ), the G-domain also contacts h44 by means of a clamp adjacent to the interaction site of h45 and h24. This clamping interaction is facilitated by the β_6,7_ hairpin and the switch I/G2 region (4); however, this hairpin is lacking in S. aureus RsgA (Fig. 5A and B), and so it is likely that the G-domain interacts with h44 singly through the switch I/G2 region.

10.1128/mBio.02679-21.2 TABLE S2 Crystallographic data and refinement statistics. Download Table S2, DOCX file, 0.01 MB. Copyright © 2021 Bennison et al. 2021 Bennison et al. https://creativecommons.org/licenses/by/4.0/ This content is distributed under the terms of the Creative Commons Attribution 4.0 International license.

10.1128/mBio.02679-21.8 FIG S6 Binding of ppGpp and the 30S ribosomal subunit by RsgA. (A) Fo-Fc/2Fo-Fc omit map of the ppGpp binding site of RsgA (blue mesh) overlaid with a stick model of the ppGpp ligand, colored as follows: carbon, orange; nitrogen, blue; oxygen, red; and phosphorous, yellow. Note the clear electron density due to the 3′-diphosphate of ppGpp. The Fo-Fc map is contoured at 3.1 σ, and the 2Fo-Fc map is contoured at 1.6 σ. (B) LigPlot maps of interacting residues involved in ppGpp binding (81). Bonds within the protein are shown in orange, whereas those within the ligand are shown in green. Hydrogen bonds are shown as pink dashed lines, with their respective bond lengths indicated (Å). Protein residues are labeled and Van der Waal’s contacts are represented as red curves. (C) Detailed views of the ppGpp binding site of RsgA, shown as stick models. RsgA residues are represented as white models, with atoms colored by type: carbon in green for the nucleotides, pale grey for the protein, nitrogen in blue, oxygen in red, and phosphorous in orange. Hydrogen bonds and electrostatic interactions between the protein and ligand are represented by yellow dashed lines. The uncommon bond length of the long-range stabilizing interaction between K116 and the ε-phosphate of ppGpp is labeled (Å). In panels B and C, specific RsgA residues are labeled, including five of the seven G1 motif residues (G167, V168, G169, K170, and S171) and two of the four G4 motif residues (K113 and D115). Download FIG S6, TIF file, 2.8 MB. Copyright © 2021 Bennison et al. 2021 Bennison et al. https://creativecommons.org/licenses/by/4.0/ This content is distributed under the terms of the Creative Commons Attribution 4.0 International license.

The ppGpp ligand is bound in an elongated conformation, where the 3′- and 5′-phosphate moieties face away from each other (see Fig. S6A). The guanosine-5′-diphosphate backbone interacts with the G-domain in an identical manner to the more well-characterized GMPPNP (see Fig. S6B and C) (4, 53), with the P-loop/G1 motif stabilizing the α,β-diphosphate and the G4 motif specifically recognizing the guanine nucleotide base. The 3′-diphosphate extends away from the core of the protein, toward the solvent and appears to be stabilized only by a long-range 5.5-Å electrostatic interaction between the lone electron pair on the ε-phosphate of ppGpp and the basic lysine residue K116 (see Fig. S6C). It is worth noting that in the GTP-bound ON state, the switch I/G2 and switch II/G3 flexible loops would aid in stabilizing both the catalytic Mg^2+^ ion and γ-phosphate (4, 53). In our structures there is no electron density corresponding to the Mg^2+^ and the switch I/G2 loop is unresolved, likely due to innate flexibility when not contacting a γ-phosphate. In addition, the switch II/G3 loop does not appear to form hydrogen-bonds or electrostatic interactions with the ligand.

### ppGpp-bound RsgA mimics the GDP-bound OFF-state conformation.

**FIG 6:**
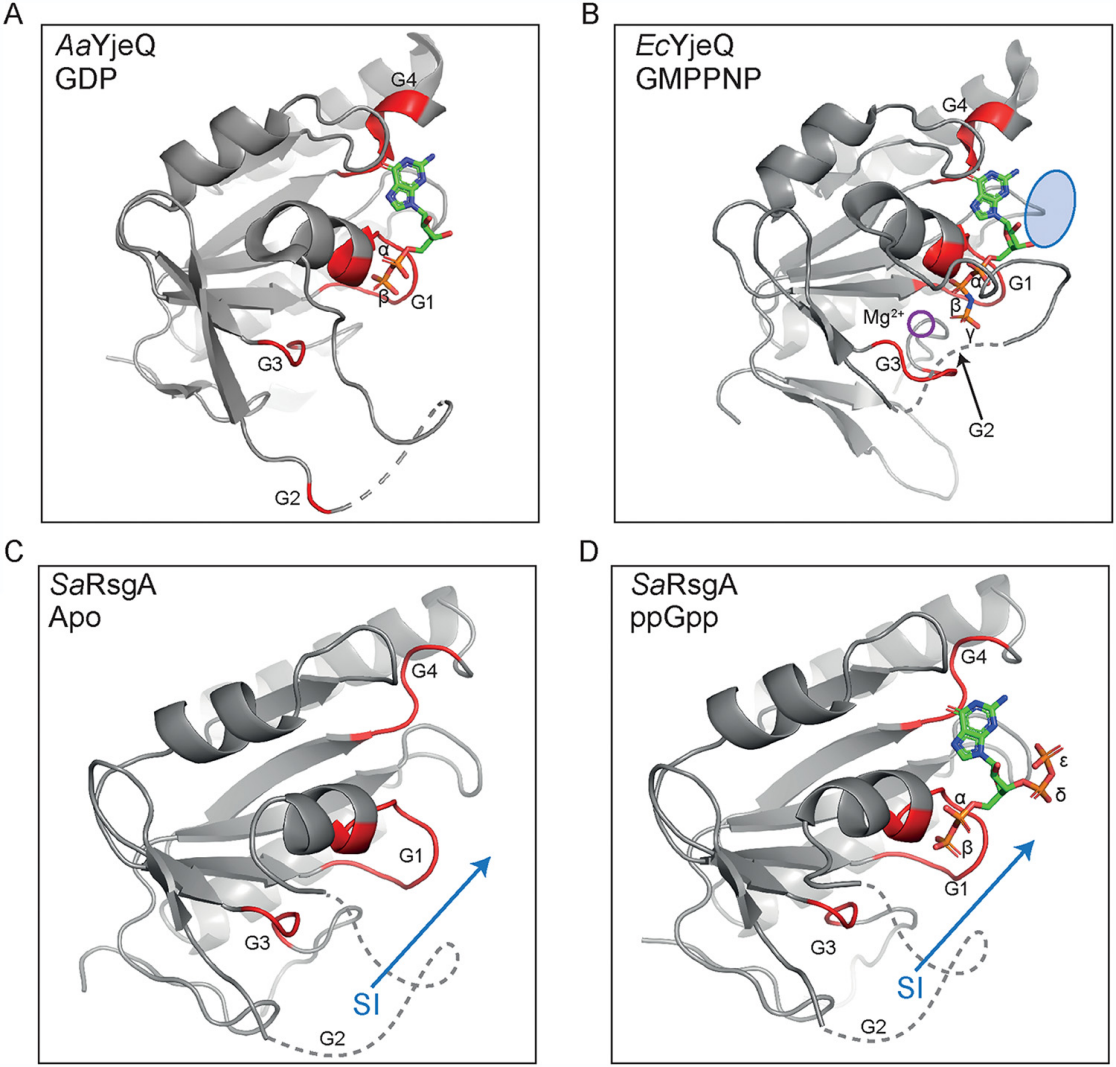
Comparison of the GTPase domains of RsgA and homologues in different nucleotide bound states. (A to D) The G-domain conformation of Aquifex aeolicus RsgA (YjeQ) bound to GDP (PDB 2YV5, chain A) (A), Escherichia coli RsgA (YjeQ) bound to GMPPNP (PDB 5UZ4, chain Z [53]) (B), Staphylococcus aureus RsgA in the apo state (PDB 6ZJO, chain A; this study) (C), and Staphylococcus aureus RsgA bound to ppGpp (PDB 6ZHL, chain A; this study) (D). RsgA/YjeQ is shown as a cartoon representation, colored gray, with the G1, G2, G3, and G4 motifs colored red where resolved. The hypothetical position of the switch I/G2 loop are represented by gray dashed lines, as determined by comparison to the resolved region of the GDP-bound YjeQ, and the bound nucleotides are colored by atom as follows: carbon, green; nitrogen, blue; oxygen, red; and phosphorous, orange. Rearrangements of the switch I/G2 loop to facilitate entry into the ON state are indicated by blue arrows. The binding site of the Mg^2+^ ion in the GMPPNP-bound conformation (B) is indicated by a purple circle, and the position of the δ,ε-phosphate of ppGpp is indicated relative to bound GMPPNP by a blue oval in panel B.

For RsgA, a catalytic histidine residue is located within the switch I/G2 loop, two residues upstream of the conserved G2 threonine (4). Therefore, correct docking of this region upon binding to either GTP or the 16S rRNA is thought to be instrumental for GTPase activity. It has also been previously proposed by Pausch et al. (6) that for RbgA, the 3′-diphosphate of (p)ppGpp prevents the movement of switch I/G2 into the ON conformation necessary for GTP hydrolysis and ribosome binding, explaining why the GTPase is incapable of hydrolyzing (p)ppGpp in a similar manner to GTP (6). In order to determine whether a similar steric inhibition is occurring for RsgA, we compared our apo- and ppGpp-bound structures with available structures of RsgA homologues, namely, Aquifex aeolicus YjeQ bound to GDP (PDB 2YV5) and E. coli YjeQ complexed with both the 30S subunit and GMPPNP (PDB 5UZ4 [53]) (Fig. 6). There is another solved structure of E. coli RsgA in the GMPPNP-bound state (PDB 5NO2), which exhibits a highly similar general GTPase domain and switch I/G2 and switch II/G3 conformation to the aforementioned GMPPNP-bound structure (PDB 5UZ4). However, upon C_α_ alignment of the GTPase domains, the bound position of GMPPNP in the PDB 5NO2 model is translated by 1.5 Å and rotated by 19° about the longitudinal *x* axis relative to the binding position of GMPPNP from the PDB 5UZ4 structure. The position and orientation of the ppGpp backbone in our structure almost perfectly reflects that of the bound GMPPNP in PDB 5UZ4, and so this model was used for comparison.

Importantly, in both of the 5UZ4 (GMPPNP-bound) and 2YV5 (GDP-bound) structures, the switch I/G2 loops were partially resolved (Fig. 6A and B). Despite a similar overall fold of the G-domain, the switch I/G2 loop in the GDP-bound structure appears to extend distally from the main body of the protein, far from the associated ligand (Fig. 6A). In contrast to this, the GMPPNP-bound structure features a fully docked switch I/G2 loop, positioned adjacent to the bound ligand and the binding site of the Mg^2+^ ion, although the Mg^2+^ ion itself is not resolved. Crucially, in this conformation, the docked switch I/G2 loop occupies the same space that the 3′-diphosphate moiety of ppGpp would (Fig. 6B and D). In addition, the switch II/G3 loop conformation differs between the GDP- and GMPPNP-bound structures, being extended toward the γ-phosphate of GMPPNP in the latter. Compared to our apo (Fig. 6C)- and ppGpp (Fig. 6D)-bound structures, the switch II/G3 region appears highly similar to that of the GDP-bound structure, leading us to conclude that the OFF conformation is maintained whether in the apo state or when bound to either GDP or ppGpp and that the switch I/G2 loop would remain disordered in the absence of GTP. This lack of docking of switch I/G2 would inhibit GTPase activity by preventing proper docking of the catalytic histidine within switch I (4), coordination of the Mg^2+^ cofactor by the G2 threonine (6), and subsequent interaction with the γ-phosphate of GTP.

### Displacement of the G2 loop by (p)ppGpp impairs RA-GTPase-ribosome interactions.

**FIG 7:**
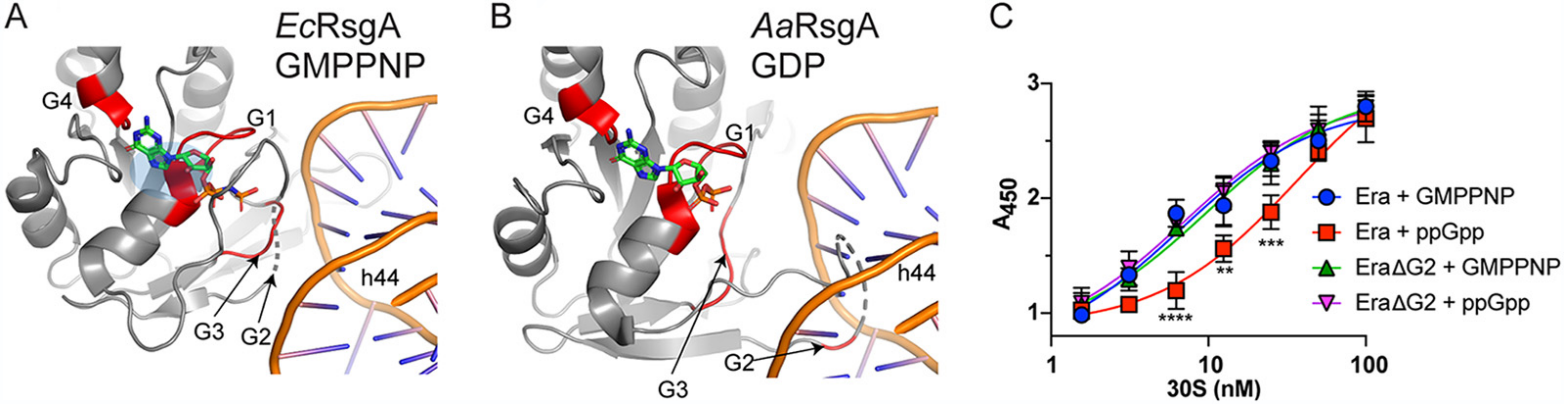
ppGpp-mediated inhibition of RA-GTPase association to ribosome subunits is facilitated by incorrect positioning of the switch I/G2 loop. (A) E. coli RsgA (YjeQ) bound to GMPPNP (PDB 5UZ4, chain Z) and chain A (16S rRNA) [53]), including a cropped view of the rRNA binding site on h44. For the full binding environment, see Fig. 5. (B) *A. aeolicus* RsgA (YjeQ) (PDB 2YV5, chain A) bound to GDP docked onto h44 of the 16S rRNA from PDB 5UZ4 (chain A) using C_α_ alignment of the G-domains. The RsgA/YjeQ is shown as a cartoon representation colored gray, with the G1, G2, G3, and G4 motifs colored red where visible. The bound nucleotides are colored by atom as follows: carbon, green; nitrogen, blue; oxygen, red; and phosphorous, orange. (C) ELISAs were carried out using 500 nM purified recombinant Era or Era ΔG2 in the presence of an excess of either GMPPNP or ppGpp and 100 nM highly pure 30S S. aureus ribosomal subunits. Bound proteins were detected using α-His HRP-conjugated antibodies, and the absorbance was quantified at 450 nm. Experiments were performed in quadruplicate, and error bars represent standard deviations (**, *P < *0.01; ***, *P < *0.001; ****, *P < *0.0001).

The structure of RsgA in the GMPPNP-bound ON state has only ever been solved when associated with the 30S ribosomal subunit, suggesting that it is stabilized in this conformation (4, 53). In order to assess the role of the switch I/G2 loop in ribosome association, we performed computational C_α_ alignments of the available GDP-bound (PDB 2YV5) structure with the GMPPNP-bound RsgA-30S ribosome complex (PDB 5UZ4) (Fig. 7A and B). It has previously been shown that each of the three domains of RsgA interact with rRNA to provide a stable docking interaction (Fig. 5C) (4) and that, for E. coli RsgA, the switch I/G2 loop and a β6,β7-hairpin clamp around h44, contacting the minor and major groove, respectively (Fig. 7A). However, when the GDP-bound OFF-state structure from *A. aeolicus* is superimposed in place of the GMPPNP structure, it appears that the switch I/G2 loop is positioned in such a way that would cause steric clashing between the phosphate backbone of h44 (Fig. 7B). Although it is important to stress that this modeling is performed using protein models and 30S subunits from separate organisms and may not perfectly represent the situation in S. aureus, this leads us to hypothesize that the misalignment of the switch I/G2 loop and subsequent steric clashing between the RA-GTPase and h44 of the 16S rRNA could be responsible for (p)ppGpp-mediated inhibition of RA-GTPase association to the ribosome.

We next sought to determine the role of the switch I/G2 loop in RA-GTPase association to the ribosome experimentally, and to this end we generated an Era ΔA38-G47 (henceforth referred to as Era ΔG2) variant lacking 10 residues of the switch I/G2 loop in line with previous studies concerning the switch I/G2 loop of E. coli Era (54). Deletion of this region had no impact on guanine nucleotide binding (see Fig. S7A), yet rendered the Era ΔG2 variant incapable of hydrolyzing GTP (see Fig. S7B). The effect of switch I/G2 loop deletion on ribosome association was determined using ELISA, where Era or Era ΔG2 were incubated with either GMPPNP or ppGpp to encourage association and dissociation from the ribosome, respectively (Fig. 7C). For the wild-type protein, the *K_d_* of Era binding to the 30S subunit decreased from 6.6 ± 2.1 nM to 36.1 ± 8.2 nM when in the ppGpp-bound state compared to the GMPPNP-bound state. However, this decrease in affinity was abolished for the Era ΔG2 variant, which was similarly capable of 30S association whether bound to GMPPNP or ppGpp, with *K_d_* values of 9.7 ± 2.4 nM and 7.1 ± 1.5 nM, respectively. The fact that ppGpp cannot reduce the affinity of Era ΔG2 to the 30S suggests that the switch I/G2 loop is essential for the alteration in ribosome association observed during the ON/OFF cycle and yet does not specifically contribute to association of the RA-GTPase to the subunit. We suggest that this region is not directly responsible for promoting subunit docking but that the switch I region instead forms electrostatic interactions with conformationally mature h44 and h45 rRNA following ribosome association, enabling positioning of the switch I/G2 loop in a catalytically active conformation. These interactions and the subsequent loop rearrangement may represent the slow stabilization step (*k*_2_) observed in our stopped flow analysis (Fig. 3).

10.1128/mBio.02679-21.9 FIG S7 Deletion of the switch I/G2 loop of Era renders the protein capable of nucleotide binding yet catalytically inactive. (A) Nucleotide binding capacity was determined using DRaCALA as described in the methods section using 10 μM of purified recombinant Era or Era ΔG2. The protein was incubated with 1.83 nM α-^32^P-labeled GTP, GDP, ppGpp, or pppGpp and then incubated for 5 min at room temperature before spotting onto nitrocellulose membrane and visualization using a phosphorimager. Experiments were carried out in triplicate, with error bars showing the standard deviations. Statistical analysis was carried out using unpaired, two-tailed *t* testing, and no significant difference was observed between the nucleotide binding capacity of Era and Era ΔG2 for GTP, GDP, ppGpp, or pppGpp. (B) 0.1 μM recombinant Era or Era ΔG2 was incubated with 0.1 μM 70S ribosomes and 1 μM cold GTP spiked with 1.83 nM α-^32^P-labeled GTP and then incubated at 37°C for 60 min. Samples were taken every 10 min, and nucleotides were separated via TLC as described in Materials and Methods. Experiments were carried out in triplicate, with error bars representing the standard deviations between repeats. Download FIG S7, TIF file, 2.9 MB. Copyright © 2021 Bennison et al. 2021 Bennison et al. https://creativecommons.org/licenses/by/4.0/ This content is distributed under the terms of the Creative Commons Attribution 4.0 International license.

## DISCUSSION

The stringent response is a multifaceted stress coping mechanism, ubiquitously used throughout the *Bacteria* to cope with nutrient starvation conditions. Recent transcriptomics data have highlighted the diversity and complexity of this response, with 757 genes being differentially regulated within 5 min of (p)ppGpp induction (25). For Gram-positive bacteria, the regulation of transcription by (p)ppGpp is intricately linked to purine nucleotide levels, which are impacted in a number of ways (55). Upon induction of the stringent response, GTP/GDP and ATP levels decrease as they are utilized by (p)ppGpp synthetase enzymes (17). Furthermore, once produced (p)ppGpp directly inhibits a number of enzymes involved in the guanylate and adenylate synthesis pathways, further reducing GTP/GDP levels (38, 56). All of this results in a shift from high GTP/GDP and low (p)ppGpp levels in fast-growing cells to low GTP/GDP and high (p)ppGpp in nutritionally starved cells. For S. aureus, the impacts of this are wide-reaching, affecting transcription initiation (39), enzyme activities (57), and—as we show here—the regulation of the activity of RA-GTPases by tuning their capacity to interact with ribosomal subunits.

### Physiological consequences of (p)ppGpp-GTPase interactions.

In the present work, we examine the nucleotide binding preferences of four RA-GTPases, and the consequences of this binding on regulating the interactions of these RA-GTPases with the ribosome. Cycling between the GTP-bound ON and GDP-bound OFF states is critically important for RA-GTPases, since it enables these proteins to act as molecular checkpoints of ribosome assembly. Here, we show that RA-GTPases bind to guanosine nucleotides competitively and with differing affinities, with GDP and ppGpp binding with up to six times greater affinity than their 5′-trinucleotide-containing counterparts GTP and pppGpp (Table 1). The consequence of differing nucleotide-bound states for interactions with ribosomal subunits is significant. We observe that the GTP-bound ON state is required to promote RsgA- and RbgA-ribosome interactions (Fig. 2 and 3). Indeed, the binding of apo RbgA to the 50S subunit was almost undetectable by stopped-flow fluorometry (Fig. 3B), although Era and HflX did demonstrate background binding to the 30S and 50S subunits by Western immunoblotting and ELISA. A cryo-electron micrograph (cryo-EM) structure of Era binding to the 30S subunit has previously been solved (12), demonstrating that this GTPase can bind in the apo form in a conformation different from either the GTP-bound or the GDP-bound states. Interestingly, the ability of the RA-GTPases to associate with the ribosomal subunit could be dependent on a canonical GTPase domain, since both circularly permuted (cp)GTPases RsgA and RbgA displayed a similar lack of apo-state association. The driving factor behind this is unclear, although it could be related to either the difference in GTPase domain orientation between the canonical and cpGTPase family or to the increased length of the switch I/G2 region in cpGTPases (58). Coupled with the difference in the response of HflX and RbgA to pppGpp observed in our stopped-flow experiments (Fig. 3), further investigation into the differential responses to stringent response alarmones between the canonical and cpGTPase families is required.

Upon induction of the stringent response, levels of (p)ppGpp in the cell rise, swiftly becoming the dominant guanosine nucleotide in the cell (17, 59), causing (p)ppGpp to outcompete GTP for occupancy of the nucleotide binding site (Fig. 1C; see also Fig. S2D to F), and resulting in reduced association of RA-GTPases to their target ribosomal subunit and reduced 70S ribosomes (Fig. 2, 3, and 4). It has been previously shown that, in contrast to our observations regarding ribosome assembly factors, ppGpp binding enhances the affinity of the (p)ppGpp-binding RA-GTPase ObgE to the 50S subunit (41). This may reflect the proposed role of ObgE as a 50S-based late-stage anti-association factor (41) that would benefit from enhanced affinity for the 50S in the ppGpp-bound state to prevent subunit joining and 70S formation. Unfortunately, we were unable to purify enough ObgE to compare using our system, although the molecular and structural mechanisms underlying this opposite effect would be interesting to investigate further.

Ribosomal rRNA production and biogenesis are not the only aspects of protein synthesis that (p)ppGpp regulates, given its ability to bind to the bacterial IF2, EF-Tu, EF-G, elongation factor Ts (EF-Ts), and release factor 3 (RF3) (21, 22, 60–63). In each case, competitive binding of (p)ppGpp to these GTPases results in an inhibition of activity and reduction of the elongation cycle. Unlike the RA-GTPases described here, both IF2 and EF-G bind to GTP, GDP, and (p)ppGpp with similar affinity (61, 62, 64), albeit with EF-G demonstrating an overall lower affinity for guanine nucleotides. Binding of the 30S pre-IC-associated IF2 to ppGpp occludes start codon recognition when bound to ppGpp-intolerable transcripts, instead promoting the association of ppGpp-tolerable transcripts such as m*TufA*, which enable GTP binding and translation to occur (60). This complex-driven reprogramming of nucleotide affinity of IF2 depends on the bound mRNA transcript. It remains a possibility that a similar system could contribute to the nucleotide-bound state of RA-GTPases while in unstable equilibrium with the ribosome or while bound to immature ribosomal subunits, with ribosome association driving nucleotide exchange to enter the GTP-bound ON state, although this seems unlikely given that the complex formation is dependent on bound GTP. With a 5-fold-higher concentration of GTP compared to GDP during exponential-phase growth in E. coli (17), the GDP/GTP binding cycle of prokaryotic TRAFAC GTPases is thought to be driven by the relative concentration-driven stochastic exchange, based on the rarity of prokaryotic guanosine exchange factors. IF2 has also been proposed to enable ribosomal subunit maturation or assembly under cold shock conditions in a GTPase-dependent fashion due to innate peptide chaperone activity; however, the effect of GTPase inhibition by (p)ppGpp on this process has not been investigated (22).

Rapid yet transient production of the alarmones ppGpp and pppGpp has been associated with the early phases of the heat shock response in B. subtilis (65), with intracellular concentrations rising in line with previously observed responses to amino acid starvation. However, (p)ppGpp concentration reduced to basal levels after 10 min of heat shock (65), and intracellular GTP levels remain relatively stable throughout, suggesting a translation-oriented response. Transcription of *hflX* has been well defined as being upregulated during heat shock (42), and the N-terminal ATP-dependent RNA helicase domain of HflX has been implicated in the repair of heat-damaged rRNA (5) and is important in enabling cell survival following heat stress. HflX is capable of dissociating the 70S complex while bound to GMPPNP, GTP, GDP, or ppGpp (13, 42), which, coupled with our data, indicates that unstable complex formation is sufficient for this subunit splitting. Prolonged complex formation and aberrant rRNA repair may occur following reduction of (p)ppGpp concentrations to a basal level (65) and reentry into the GTP-bound state. Further investigation into precise timings of HflX-mediated 70S splitting, 100S splitting, and rRNA helicase activity during the heat shock response are required to further understand the role of the ppGpp-mediated reduction of ribosome association in this context.

### Structural consequences of (p)ppGpp-GTPase interactions.

The biochemical studies carried out here indicate that ppGpp-bound RsgA most likely mimics the GDP-bound OFF state (Fig. 2 and 3), an assertion that we corroborate by solving the crystal structure of RsgA in the apo- and ppGpp-bound states (Fig. 5 and 6). These structures are in line with previous crystallographic studies regarding the different nucleotide-bound conformations of RbgA (6). In each case, (p)ppGpp was shown to inhibit GTPase activity through displacement of the switch I/G2 loop into an OFF-state conformation, which our computational alignments demonstrate is incompatible with stable interaction with the ribosome subunit (Fig. 7). Given the reaction scheme determined by stopped-flow fluorescence, it is possible that the slower stabilization step (*k*_2_) observed when HflX is in the ppGpp-bound state compared to the GTP-bound state could be due to improper loop docking following association of the RNA-binding domain(s) with the ribosome, leading to dissociation. In the E. coli homologue of RsgA, the switch I/G2 loop contacts the minor groove of h44 to facilitate entry into the active conformation (4), whereas the lack of docking in the A. aeolicus and S. aureus GDP and ppGpp-bound models suggests a steric hindrance to association. Indeed, deletion of the switch I/G2 loop of S. aureus Era was shown to have no impact on nucleotide binding or 30S subunit association, while completely abrogating the GTPase activity (Fig. 7C; see also Fig. S7). The loss of inhibition of ribosome binding when in the ppGpp-bound state suggests that the switch I/G2 loop is not a specific mediator of association, and instead represents a steric hindrance to complex stabilization while in the OFF state, suggesting a regulatory mechanism which could be common among other RA-GTPases or GTPases in general.

Similar to our RsgA-ppGpp structure, the diphosphate moieties of ppGpp bound by RbgA are in the elongated conformation (6), where the 3′- and 5′-phosphate moieties face away from each other. This configuration is not consistent among all (p)ppGpp-binding proteins or even among RA-GTPases. For example, the E. coli RA-GTPases BipA and ObgE bind to ppGpp in a ring-like conformation (66–68), in which the 3′ and 5′ phosphate moieties point toward each other. While no structural reasoning for this difference in conformation is known, aside from to extend the breadth of responses controlled by (p)ppGpp, it has been suggested that proteins that bind (p)ppGpp in the ring-like conformation have 10-fold-lower inhibitory constants and dissociation constants than those which bind in the elongated conformation (69, 70). This could potentially influence the temporal or energetic threshold during the stringent response where a certain protein becomes inhibited, based on decreasing concentrations of GTP and increasing concentrations of (p)ppGpp (19, 38).

### Conclusion.

**FIG 8:**
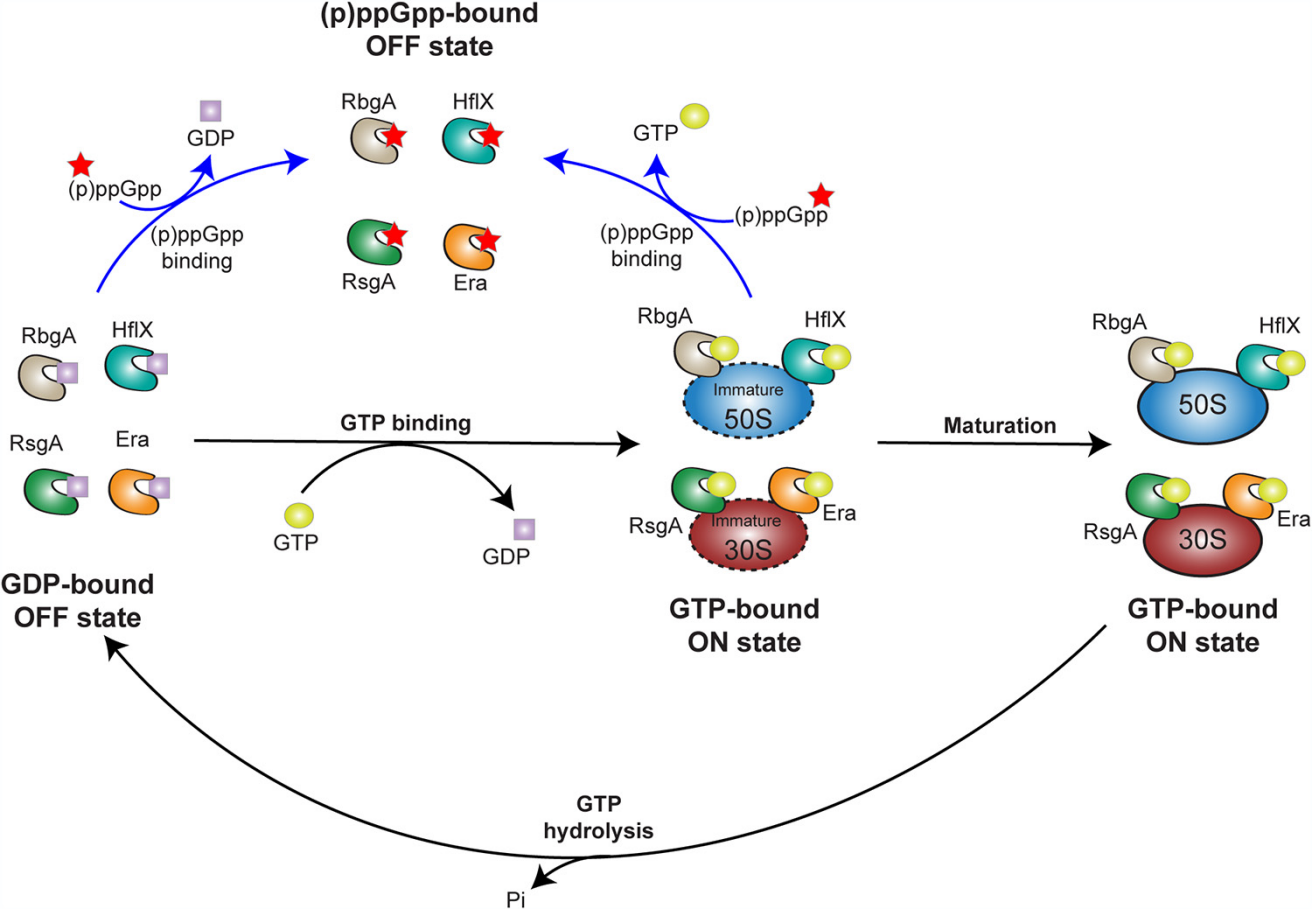
Model of the control of ribosome maturation by (p)ppGpp and RA-GTPases. Under proliferative conditions, GTP binds to RA-GTPases, enabling association to the immature ribosome subunits and subsequent maturation, at which point GTP is hydrolyzed to GDP and the RA-GTPase dissociates from the ribosome. Under stringent conditions (blue arrows) when cellular (p)ppGpp concentrations rise and GTP and GDP concentrations fall (38, 79), (p)ppGpp can outcompete GTP or GDP for RA-GTPase binding. This inhibits GTPase activity and destabilizes the association of RA-GTPases to the ribosome subunits and negatively impacts ribosome biogenesis.

Altogether, our data favor a model (Fig. 8) whereby in unstressed growing cells, GTP is the predominant nucleotide and induces the RA-GTPase ON-state conformation. Binding of the enzymes to each individual ribosomal subunit follows in order to promote a processing event. Following this, GTP is hydrolyzed to GDP, with the free energy of hydrolysis inducing transition to the OFF state and subsequent dissociation. Upon cell starvation, the concentration of (p)ppGpp in the cell rises sharply, where it can outcompete GTP for binding to the RA-GTPases. The increase in (p)ppGpp not only inhibits the GTPase activity but also negatively impacts the stability of RA-GTPase–ribosome interactions, reducing biogenesis and slowing growth. With the rapid accumulation and high affinity of interaction, it is likely that inhibition of RA-GTPases by (p)ppGpp could occur extremely early during the stringent response, rapidly halting the *de novo* production of ribosomal subunits (19). Furthermore, due again to this high affinity, (p)ppGpp could remain bound to RA-GTPases, preventing further ribosome biogenesis during low-energy conditions, yet preserving a pool of enzymes ready for rapid resumption of growth upon restoration of the proliferative state. Another distinct possibility is that, due to basal concentration of (p)ppGpp being remarkably similar to the binding affinity of these RA-GTPases (19), general regulation of ribosomal production could also be in part controlled by this alarmone—perhaps by enabling the cell to respond to slight fluctuations in the GTP pool in the absence of an overall stringent response.

Here, we have used complementary techniques to demonstrate that (p)ppGpp prevents stable association of RA-GTPases to the ribosome, both *in vitro* and within the bacterial cell. While there may be differing affinities between the enzymes, this is achieved overall by these proteins having a stronger affinity for ppGpp over GTP, with ppGpp interactions holding these enzymes in an OFF-state conformation. Consequently, this imparts delays to 70S ribosome assembly, which in turn contributes to the growth defects that are observed upon induction of the stringent response. Altogether, we highlight RA-GTPase–(p)ppGpp interactions as important regulators of stringent response-mediated growth control.

## MATERIALS AND METHODS

### Bacterial strains and culture conditions.

E. coli strains were grown in Luria-Bertani broth (LB) and S. aureus strains in tryptic soy broth (TSB) at 37°C or 30°C with aeration. Strains are listed in Table S1 in the supplemental material. Antibiotics were used when appropriate at the following concentrations: kanamycin, 30 μg/ml; chloramphenicol, 7.5 μg/ml (unless otherwise stated); carbenicillin, 50 μg/ml; spectinomycin, 250 μg/ml; and tetracycline, 2 μg/ml. pCN55iTET-*era*-His (iTET-*era*) was constructed by amplifying *era* from LAC* genomic DNA and cloning into the KpnI/SacI sites of pCN55iTET (iTET). pALC2073-*relP* was created by amplifying *relP* from S. aureus strain LAC* and cloning into the KpnI/SacII sites of pALC2073. Era lacking the G2 loop from amino acids 38 to 47 was constructed by splicing overlap extension PCR using LAC* genomic DNA as a template before cloning into pET28b. All plasmids were initially transformed into E. coli strain XL1-Blue, and the sequences of all inserts were verified by fluorescence automated sequencing by GATC. For protein expression and purification, all pET28b derived plasmids were transformed into E. coli strain BL21(DE3). All S. aureus plasmids were first electroporated into RN4220 Δ*spa* before isolation and electroporation into USA300 strains.

10.1128/mBio.02679-21.1 TABLE S1 Bacterial strains used in this study. Download Table S1, DOCX file, 0.01 MB. Copyright © 2021 Bennison et al. 2021 Bennison et al. https://creativecommons.org/licenses/by/4.0/ This content is distributed under the terms of the Creative Commons Attribution 4.0 International license.

### GTPase assays.

GTPase activity assays were performed as previously described (10). Briefly, the ability of proteins to hydrolyze GTP was determined by incubating 100 nM recombinant protein with 100 nM S. aureus 70S ribosomes, 1 μM GTP and 2.78 nM [α-^32^P]GTP in 40 mM Tris (pH 7.5), 100 mM NaCl (100 mM KCl for RbgA), and 10 mM MgCl_2_ at 37°C for the indicated times. For GTPase time courses, reactions were set up as described above but 5-μl samples were taken at the indicated time points. All reactions were also set up in the absence of enzymes to monitor spontaneous GTP hydrolysis. Reactions were heat inactivated at 95°C for 5 min to precipitate proteins and release bound nucleotide. Precipitated proteins were pelleted by centrifugation at 17,000 × *g* for 10 min. Reaction products were visualized by thin-layer chromatography (TLC) in PEI cellulose TLC plates (Macherey-Nagel) and separated using 0.75 M KH_2_PO_4_ (pH 3.6) buffer. The radioactive spots were exposed to a BAS-MS imaging plate (Fujifilm) and visualized using an LA 7000 Typhoon PhosphorImager (GE Healthcare), and images were quantified using ImageQuant (GE Healthcare).

### Synthesis of ^32^P-labeled (p)ppGpp and DRaCALA.

The synthesis of (p)ppGpp and differential radial capillary action of ligand assay (DRaCALA) binding and competition assays were performed as described previously (27), using recombinant, 6×His-tagged protein at the concentrations specified in the figure legends.

### Protein purifications.

Proteins were purified from 1- to 2-liter E. coli BL21(DE3) cultures. Cultures were grown at 37°C to an optical density at 600 nm (OD_600_) of 0.5 to 0.7, and expression was induced with 1 mM IPTG (isopropyl-β-d-thiogalactopyranoside), followed by incubation for 3 h at 30°C. Cell pellets were resuspended in 5 ml of buffer A (50 mM Tris [pH 7.5], 150 mM NaCl, 5% glycerol, 10 mM imidazole) and lysed by sonication upon addition of 20 μg/ml lysozyme and 30 μg/ml RNase A. Protein purifications were performed by nickel affinity chromatography. The filtered cell lysate was loaded onto a 1-ml HisTrap HP Ni^2+^ column (GE Healthcare) before elution using a gradient of buffer B (50 mM Tris [pH 7.5], 200 mM NaCl, 5% glycerol, 500 mM imidazole). Protein-containing fractions were dialyzed in 50 mM Tris-HCl (pH 7.5)–200 mM NaCl–5% glycerol before concentration using a 10-kDa centrifugal filter (Thermo Scientific) and storage at –80°C. Protein for use in crystallography was dialyzed into 25 mM Tris-HCl (pH 7.5)–200 mM NaCl and used immediately. Protein concentrations were determined by absorbance at 280 nm using appropriate extinction coefficients. *A*_260_/*A*_280_ ratios were monitored to ensure that preparations had low RNA/nucleotide contamination (<5%), indicated by a ratio below 0.8. The extinction coefficients at 280 nm for each protein and their mutant variants were calculated from the primary structure: Era, 25,900 M^−1 ^cm^−1^; RsgA, 23505 M^−1 ^cm^−1^; RbgA, 40,910 M^−1 ^cm^−1^; and HflX, 24,870 M^−1 ^cm^−1^. Typically, protein purity was above 95%, as assayed by 12% SDS-PAGE and Coomassie blue staining.

### 30S, 50S, and 70S ribosome purification.

70S ribosomes were purified as described previously (27), with the following exceptions: after purification of mature 70S ribosomes, the ribosome pellet was resuspended in dissociation buffer (20 mM Tris [pH 7.5], 120 mM NH_4_Cl, 1.5 mM MgCl_2_, 2 mM β-mercaptoethanol) and quantified using the absorbance at 260 nm, as described previously (40). A total of 50 *A*_260_ U of 70S ribosomes was applied to a 10 to 40% continuous sucrose gradient made up in dissociation buffer and separated at 111,000 × *g* for 16 h. Gradients were fractionated by upward displacement of 250-μl aliquots, which were analyzed for RNA content at an absorbance of 260 nm. Fractions containing 30S and 50S ribosomal subunits were pooled separately, and purification was continued as described previously (40).

### *In vitro* ribosome association assays.

A 500 nM concentration of recombinant 6×His-tagged RA-GTPase was incubated at room temperature for 5 min with 200 nM S. aureus 70S ribosomes in dissociation buffer (20 mM Tris [pH 7.5], 120 mM NH_4_Cl, 1.5 mM MgCl_2_, 2 mM β-mercaptoethanol) in the apo form and in the presence of 40 μM GTP, GMPPNP, GDP, ppGpp, or pppGpp. The resultant reaction (150 μl) was layered onto a 10 to 40% continuous sucrose density gradient in dissociation buffer. Subsequently, gradients were centrifuged for 16 h at 111,000 × *g* in order to separate the 30S and 50S subunits. Gradients were fractionated by upward displacement of 250-μl aliquots, which were analyzed for RNA content at an absorbance of 260 nm. Fractions containing 30S and 50S ribosomal subunits were pooled separately, and the protein content was precipitated by the addition of 10% (vol/vol) trichloroacetic acid, followed by incubation for 3 h at 4°C. Samples were centrifuged at 17,000 × *g* for 5 min and washed twice with ice-cold acetone prior to drying of the pellets at 37°C for 10 min. Pellets were resuspended in 2× SDS-PAGE sample buffer (62.5 mM Tris-HCl [pH 6.8], 2% SDS, 10% glycerol, 0.01% bromophenol blue, 10% [vol/vol] β-mercaptoethanol), and proteins were separated using a 10% SDS-PAGE gel and transferred onto a polyvinylidene difluoride Immobilon-P membrane (Merck Millipore). The membrane was blocked with 5% (wt/vol) milk in TBST (50 mM Tris-HCl [pH 7.6], 150 mM NaCl, 0.1% Tween 20), probed using 1:500 monoclonal anti-His horseradish peroxidase (HRP)-conjugated antibodies (Sigma), and imaged using a ChemiDoc MP (Bio-Rad). Band densitometry was performed using ImageJ.

### Growth and *in vivo* ribosome association assays.

S. aureus strains were grown overnight in TSB containing the appropriate antibiotics. For growth curves, overnight cultures were diluted to a starting OD_600_ of 0.05 in the presence of 100 ng/ml anhydrotetracycline (Atet) and appropriate antibiotics and grown at 37°C with aeration, with OD_600_ values determined at 2-h intervals. For ribosome association assays, cultures of USA300 Δ*era* iTET-*era*-His, USA300 (p)ppGpp-null Δ*era* iTET-*era*-His, USA300 Δ*era* iTET-*era*-His pALC2073, and USA300 Δ*era* iTET-*era*-His pALC2073-*relP* were diluted to an OD_600_ of 0.05 in fresh TSB supplemented with the appropriate antibiotics and grown to an OD_600_ of 0.6 to 0.8 before induction with 100 ng/ml Atet for 30 min. USA300 Δ*era* iTET-*era*-His and USA300 (p)ppGpp-null Δ*era* iTET-*era*-His strains were further induced with 0.05 or 60 μg/ml mupirocin for 15 min. All cultures were then shocked with 100 μg/ml chloramphenicol for 3 min before gently cooling them to 4°C to produce runoff ribosomes. Cells were centrifuged at 4,000 × *g* for 10 min, and pellets were resuspended to an OD_600_ of 35 in dissociation buffer (20 mM Tris [pH 7.5], 120 mM NH_4_Cl, 1.5 mM MgCl_2_, 2 mM β-mercaptoethanol). Cells were lysed by the addition of 0.5 μg/ml lysostaphin and 75 ng/ml DNase for 60 min at 37°C. Lysates were centrifuged at 17,000 × *g* for 10 min to remove cell debris, and 250 μl of the lysate was layered onto a 10 to 40% continuous sucrose gradient in dissociation buffer. Subunit separation was continued according to the *in vitro* method. After separation, 30S- and 50S-containing fractions were pooled and normalized using the absorbances at 260 nm to 0.65 and 0.85, respectively, to ensure equal loading in terms of ribosome content, such that associated proteins could be compared. Associated C-terminally histidine-tagged Era (Era-His) was quantified via Western blotting and band densitometry (ImageJ). Crude lysates were loaded alongside pulled-down protein to verify the Era-His expression level. Staining of the blotting membrane with Ponceau-S in 5% acetic acid was used to ensure consistent lysate loading following visualization of the immunoblot and to ensure equal ribosomal content between samples. Membranes were incubated with staining solution for up to 5 min and washed with distilled water until the background was clear.

### Ribosome profiles from *S. aureus* cell extracts.

Crude isolations of ribosomes from S. aureus cell extracts were achieved as described by Loh et al. with some modifications (45). Briefly, 100-ml cultures of the different S. aureus strains were grown to an OD_600_ of 0.4 in TSB medium with 100 ng/ml Atet. Chloramphenicol (100 μg/ml) was added to each culture, followed by incubation for 3 min, before being cooled to 4°C to enhance the pool of 70S ribosomes. Pelleted cells were suspended in association buffer (20 mM Tris-HCl [pH 7.5], 8 mM MgCl_2_, 30 mM NH_4_Cl, 2 mM β-mercaptoethanol) and normalized to an OD_600_ of 15. Cells were lysed by the addition of 0.2 μg/ml lysostaphin and 75 ng/ml DNase, followed by incubation for 60 min at 37°C. Cell debris was removed by centrifugation at 17,000 × *g* for 10 min. Clarified lysates (250 μl) were layered onto 10 to 50% discontinuous sucrose density gradients made in association buffer. Gradients were centrifuged for 7 h at 192,100 × *g*. Gradients were fractionated by upward displacement of 250-μl aliquots, which were analyzed for RNA content by determining the absorbance at 260 nm.

### Crystallization of RsgA.

The purified recombinant protein consisted of 311 residues, comprising 291 residues of S. aureus RsgA with an N-terminal 20 residue tag: MGSSHHHHHHSSGLVPRGSH. It was simultaneously buffer exchanged into 25 mM Tris-HCl (pH 7.5)–200 mM NaCl buffer and concentrated to 30 mg/ml for crystallization screening using the sitting-drop vapor diffusion method. Each droplet contained 200 nl of protein solution and 200 nl of crystallization reagent from an adjacent well (50 μl [volume]). Figures were prepared in PyMOL (The PyMOL Molecular Graphics System, version 2.0; Schrödinger, LLC), with the exception of electron density maps, which were generated using COOT (71, 72).

### RsgA-ppGpp.

The concentrated RsgA solution was supplemented with 2 mM MgCl_2_ and 2 mM ppGpp. Successful crystallization was observed when this sample was mixed 1:1 with well solution containing 0.2 M sodium citrate tribasic dihydrate, 0.1 M Bis-Tris propane (pH 6.5), and 20% (wt/vol) PEG 3350, followed by incubation at 17°C. Rod-shaped crystal clusters appeared after a few days. Crystals were transferred to a cryoprotectant solution consisting of mother liquor with 15% ethylene glycol added and flash cooled in liquid N_2_. X-ray diffraction data were collected from a single crystal on beamline i04 at the Diamond Light Source national synchrotron facility at a wavelength of 0.97949 Å. The ppGpp-bound crystals diffracted to a resolution of 1.94 Å (PDB 6ZHL). Initial processing was completed using the Xia2 pipeline (73). The crystals belonged to the space group P2_1_2_1_2_1_ (see Table S2). The structure of RsgA-ppGpp was solved via molecular replacement, using the previously published Bacillus subtilis homologue YloQ (PDB 1T9H) as a model. The structure contained one RsgA monomer in the asymmetric unit. Molecular replacement was carried out using Phaser from within the CCP4 suite (74, 75). The structure was refined via rounds of manual model building and refinement using COOT (72) and REFMAC5 (76). The final model was validated using MOLPROBITY (77). Residues 181 to 200 lacked electron density and, as such, were omitted from the final model.

### apo RsgA.

Crystallization of apo RsgA was achieved when the concentrated protein sample was mixed 1:1 with well solution containing 0.15 M ammonium sulfate, 0.1 M MES (pH 6.0), and 15% (wt/vol) PEG 4000, followed by incubation at 17°C. A single rod-shaped crystal formed after a few weeks and diffracted to a 2.01-Å resolution (PDB 6ZJO). Initial processing was completed using the Xia2 pipeline, and the crystal belonged to the space group P12_1_1 (see Table S2). The structure was solved via molecular replacement as described above, using the available RsgA-ppGpp structure as a model with ligands removed, and contained two RsgA monomers in the asymmetric unit. Iterative rounds of modeling, refinement, and validation were carried out as described above. Residues 180 to 200 (chain A) and 179 to 200 (chain B) lacked electron density and, as such, were omitted from the model.

### Fluorescent labeling of proteins.

Recombinant protein (200 μM) was incubated with 5 mM dithiothreitol (DTT) for 1 h at room temperature. DTT was removed via two consecutive passes through a PD-10 Sephadex G-25 M buffer exchange column (GE Healthcare) according to the manufacturer’s instructions into labeling buffer (50 mM HEPES [pH 7.1], 200 mM KCl, 5% glycerol, 120 μM TCEP). Flowthrough was analyzed for protein content at 280 nm. Reduced protein (50 μM) was incubated with 100 μM ATTO 488-maleimide (ATTO-TEC) overnight at 4°C, shielded from light, and subjected to gentle shaking. The reaction was stopped by the addition of 6 mM β-mercaptoethanol, and mixtures were applied to a 1-ml HisTrap HP Ni^2+^ column (GE Healthcare) before elution using a gradient of buffer B (50 mM Tris [pH 7.5], 200 mM NaCl, 5% glycerol, 500 mM imidazole) and subsequent dialysis to remove the imidazole. Labeling efficiency was calculated in accordance with the fluorescent dye manufacturer’s guidelines.

### Stopped-flow fluorescence kinetics measuring ribosome association.

For initial controls, 0.2 μM Atto-488-labeled proteins were rapidly mixed with 0.2 μM E. coli 50S ribosomal subunits (purified as described elsewhere [78]) in TAKM7 buffer (25 mM Tris-HCl [pH 7.4], 70 mM ammonium acetate, 30 mM KCl, 7 mM MgCl_2_) using an SX20 stopped-flow apparatus (Applied Photophysics) in the presence or absence of GTP, ppGpp, and pppGpp. Equal volumes (60 μl) of each reactant were rapidly mixed at 25°C. Atto-488 was excited using a 470-nm LED and fluorescence was detected through a 515-nm long-pass filter. Reactions were monitored for 10 s, with 1,000 total data points per reaction. Each condition was subject to at least five technical repeats, with curves representing the mean average fluorescence of the technical repeats.

For titrations, 0.075 μM RbgA or 0.05 μM HflX labeled proteins were mixed with a 200-fold excess of GTP, ppGpp, or pppGpp (15 μM for RbgA and 10 μM for HflX) in TAKM7 buffer just prior to use. E. coli ribosomal 50S subunits were used in excess relative to the labeled protein in the presence of nucleotides at up to 0.8 μM. Samples were then loaded separately into an SX20 stopped-flow apparatus. Equal volumes (60 μl) of each reactant were rapidly mixed at 25°C, and the fluorescence emission was monitored as described above. The resultant fluorescence time courses were fitted using the double exponential function $F=F_{0}+(A_{1}\times e^{{-k}_{\text{app}1}t})+\left(A_{2}\times e^{{-k}_{\text{app}1}t}\right)\;$ with the fluorescence signal at time *t* (*F*), the initial fluorescence signal (*F*_0_), the amplitude of signal change of the first exponential (*A*_1_), the apparent rate of the first exponential (*k*_app1_), the amplitude of signal change of the second exponential (*A*_2_), the apparent rate of the second exponential (*k*_app2_), and time (*t*). Each time course was fitted individually, with curves shown representing the mean average of at least five technical replicates. If necessary, a linear term was included. Data were normalized to the mean of the first 10 fluorescence measurements. The microscopic constants *k*_1_, *k*_–1_, *k*_2_, and *k*_–2_ were calculated by plotting both the sum and product of the apparent rates *k*_app1_ and *k*_app2_ for each titration and analyzing the resulting linear relationship using linear regression. Briefly, taking *A* as the linear regression of the sum of *k*_app1_ and *k*_app2_, and *B* as the linear regression of the product of *k*_app1_ and *k*_app2_, kinetic parameters were determined as follows:

$$k_{1}=slope(A),$$

$$k_{-1}=intercept\left(A\right)-\frac{slope(B)}{slope(A)},$$

$$k_{2}=intercept\left(A\right)-k_{-1}-k_{-2},$$

and

$$k_{-2}=\frac{intercept(B)}{k_{-1}}.$$

Dissociation constants (*K_d_*) were calculated using the following equation:

$$K_{d}=\frac{\left(\frac{k_{-1}\times k_{-2}}{k_{1}}\right)}{\left(k_{-2}+k_{2}\right)}.$$

### ELISA.

Doubling dilutions of purified S. aureus 30S ribosomes starting at 100 nM in a final volume of 100 μl in TAKM7 (5 mM Tris-HCl [pH 7.4], 70 mM ammonium acetate, 30 mM KCl, 7 mM MgCl_2_) were left static at 4°C for 16 h to coat the wells. The plates were washed three times using PBST (10 mM phosphate buffer [pH 7.4], 137 mM NaCl, 2.7 mM KCl, 0.1% Tween 20) and blocked using 5% (wt/vol) bovine serum albumin (BSA) in PBST for 2 h at room temperature. After blocking, 100 μl of 500 nM His-tagged protein made up in TAKM7 plus 5% (wt/vol) BSA was added to each well, followed by incubation statically at room temperature for 1 h. Wells were washed three times as described above, and 100 μl of anti-His HRP-conjugated antibodies (Sigma), diluted 1:10,000 in TAKM7 plus 5% (wt/vol) BSA, was added, followed by incubation at room temperature for 1 h. Wells were washed three times and developed using 100 μl 3,3′,5,5′-tetramethylbenzidine for up to 10 min until the color developed. Development was stopped and fixed by the addition of 0.67 M H_2_SO_4_, and association of the protein to ribosomal subunits was quantified by measuring the absorbance at 450 nm in a Sense 425-301 microplate reader (Hidex). Control wells were included for each protein tested, lacking either ribosomal subunits or test protein, to check for cross-reactivity.

### Statistics.

Statistical analyses were performed using GraphPad Prism 8.0 software. Statistical differences between samples were assessed using either two-tailed, unpaired *t* testing or one-way analysis of variance (ANOVA), followed by Tukey’s multiple-comparison test, as indicated in the figure legends.

### Data availability.

The coordinates and electron density maps of RsgA-apo and RsgA-ppGpp have been deposited in the Protein Data Bank in Europe (PDBe; https://www.ebi.ac.uk/pdbe/node/1) under accession codes 6ZJO and 6ZHL, respectively.
