# Supplementary figures and images for: The Stringent Response Inhibits 70S Ribosome Formation in Staphylococcus aureus by Impeding GTPase-Ribosome Interactions
Source: mBio. 2021 Nov 9;12(6):e02679-21. doi: 10.1128/mBio.02679-21 (PMC8579695; doi:10.1128/mBio.02679-21)

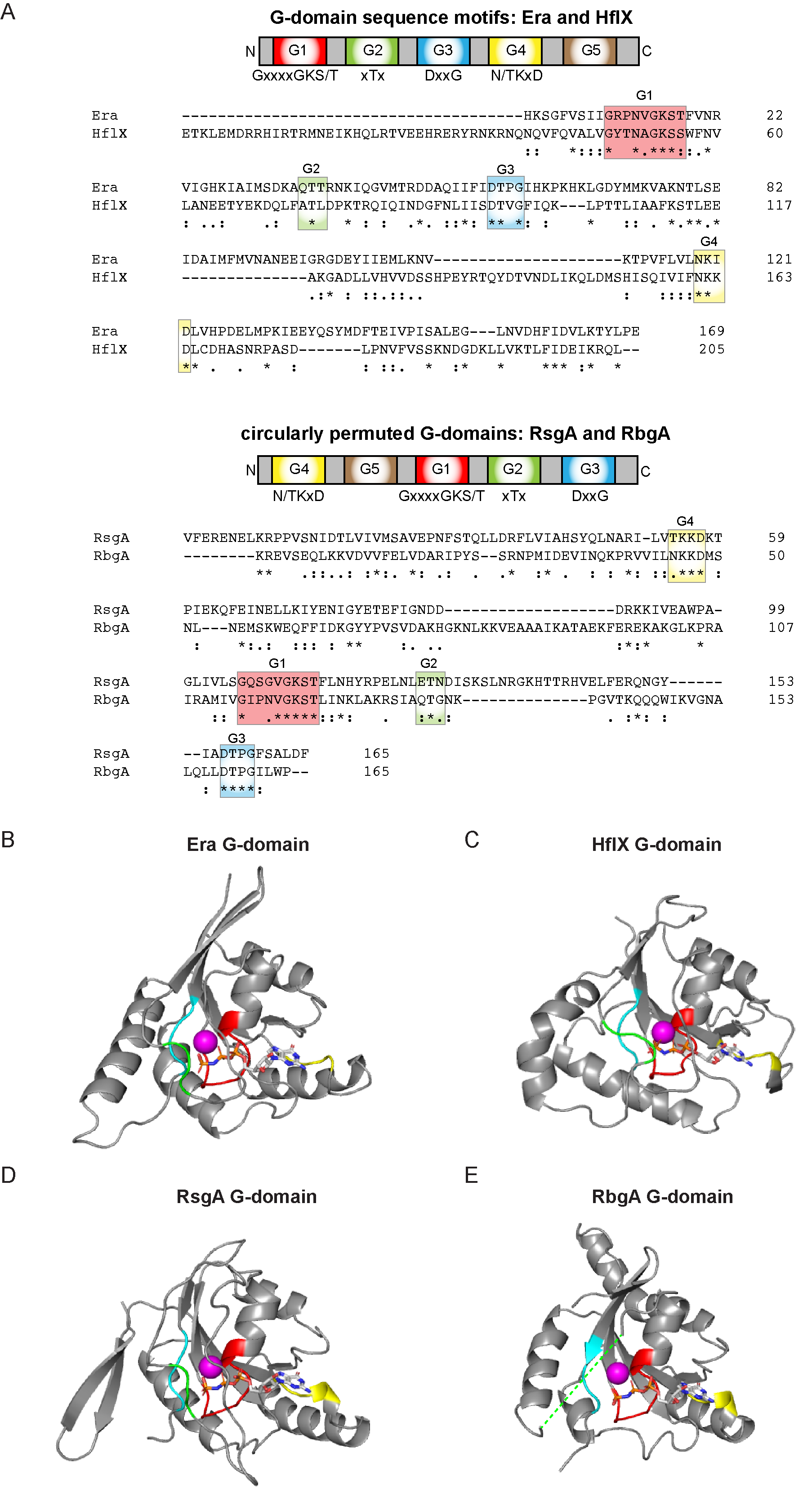

Supplement: FIG S1 [file mbio.02679-21-sf001.tif]

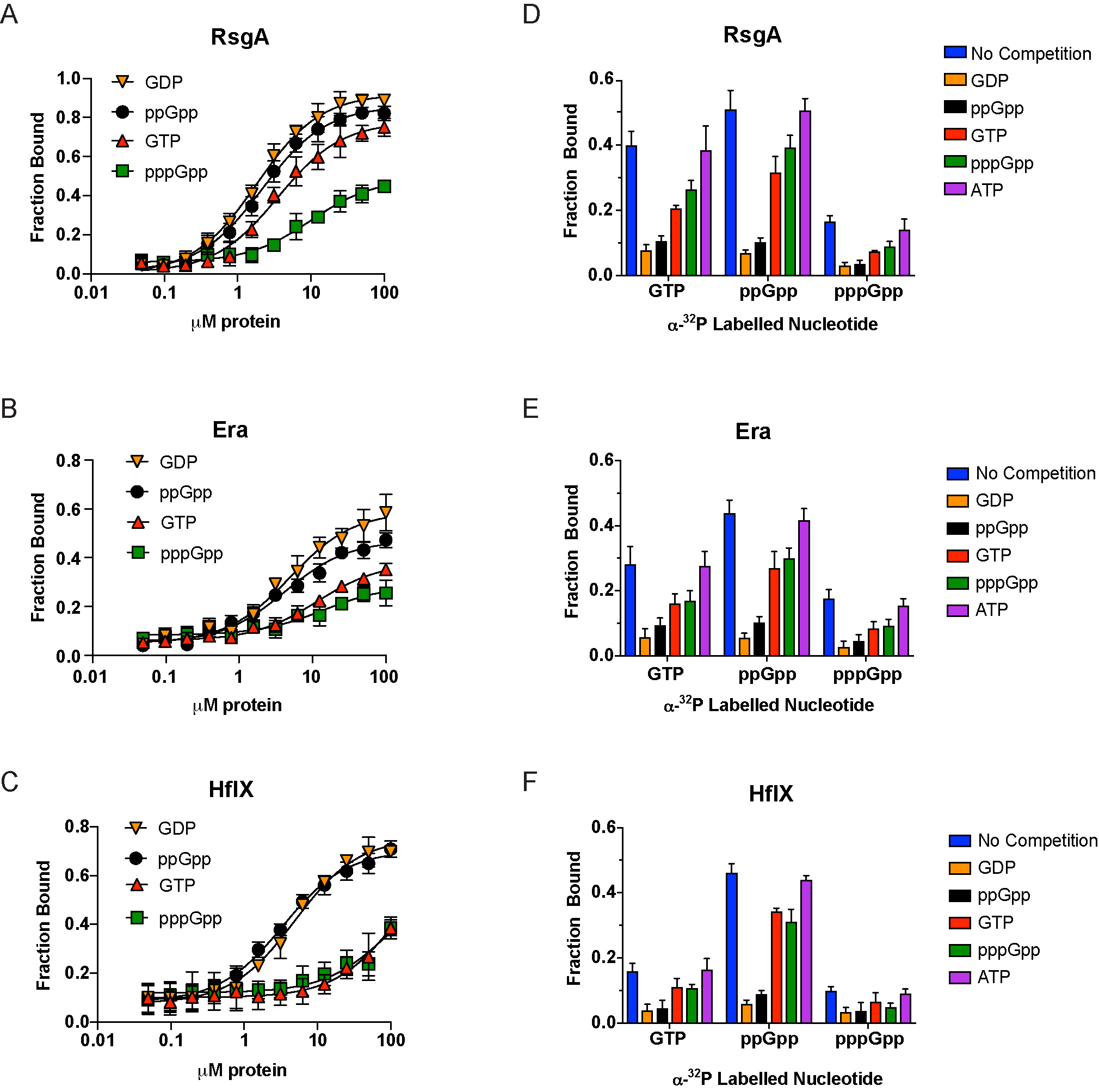

Supplement: FIG S2 [file mbio.02679-21-sf002.tif]

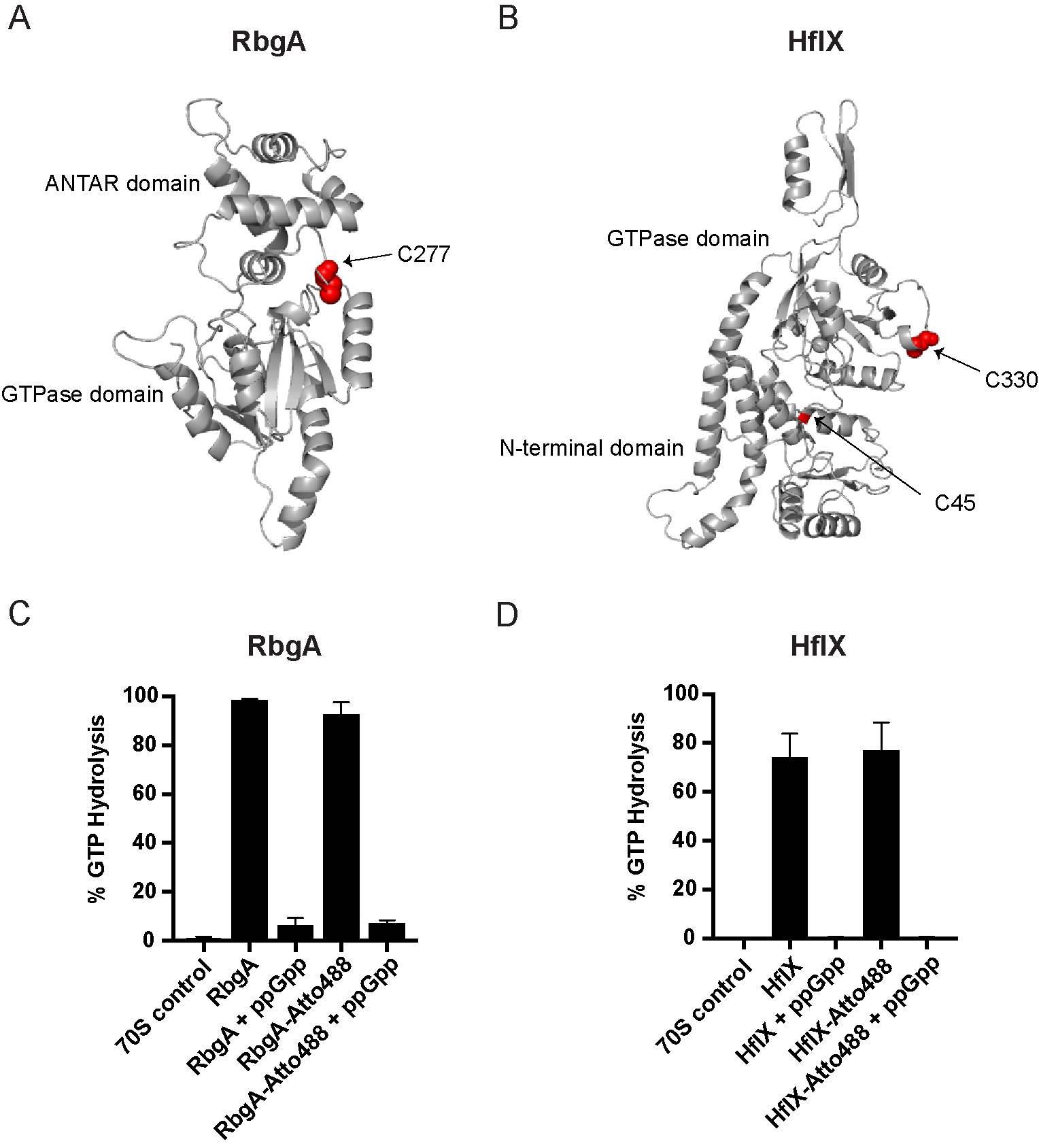

Supplement: FIG S3 [file mbio.02679-21-sf003.tif]

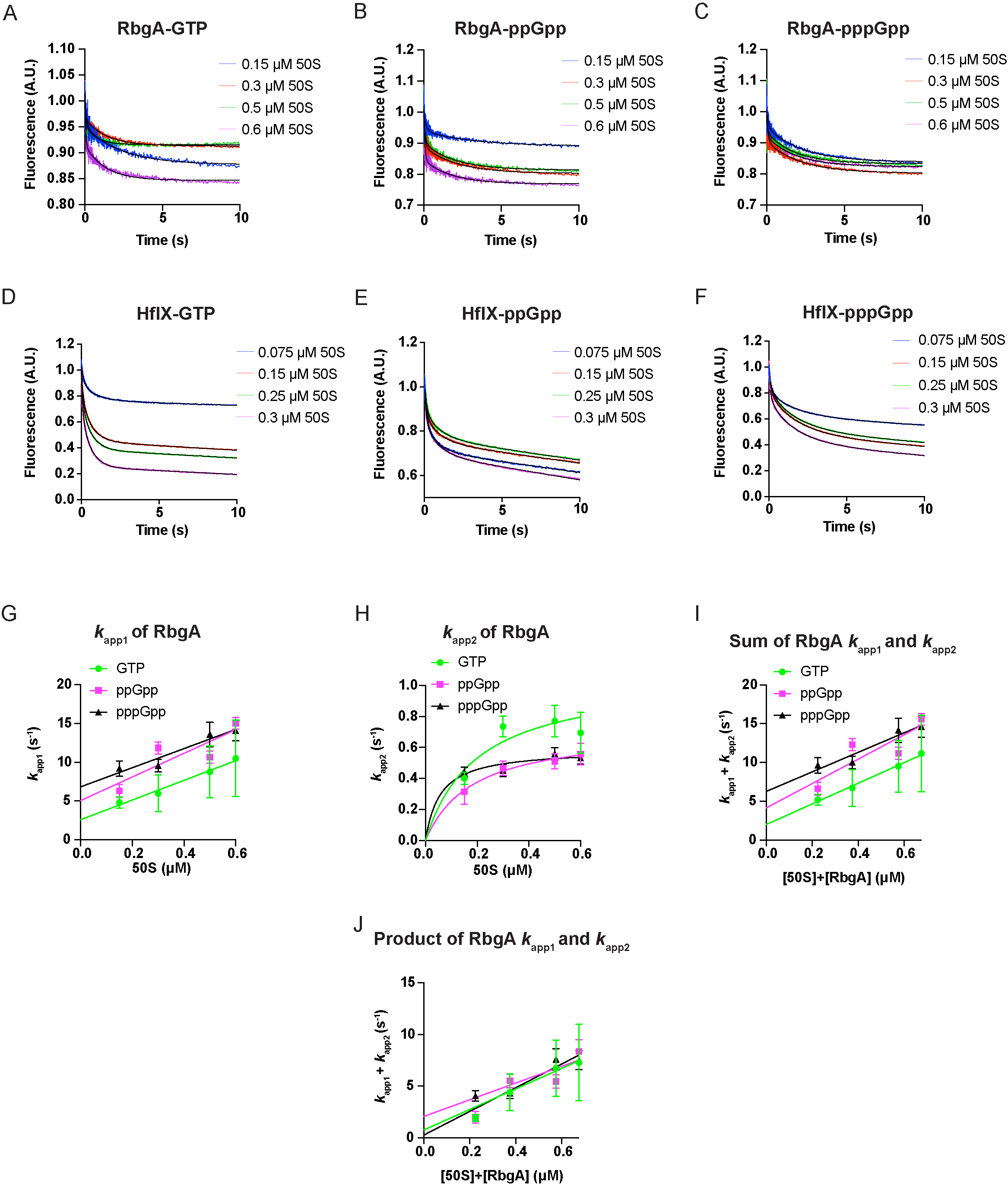

Supplement: FIG S4 [file mbio.02679-21-sf004.tif]

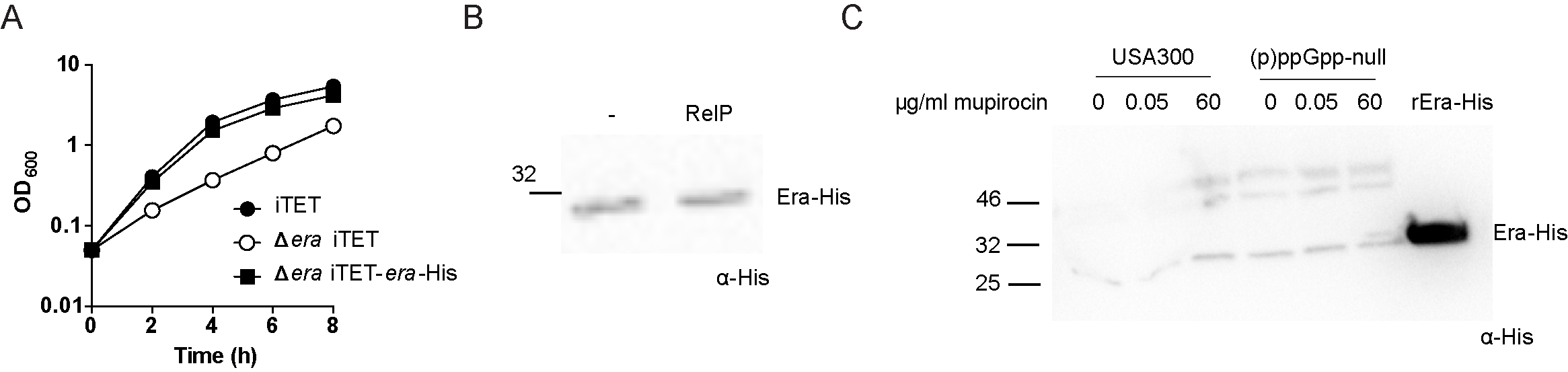

Supplement: FIG S5 [file mbio.02679-21-sf005.tif]

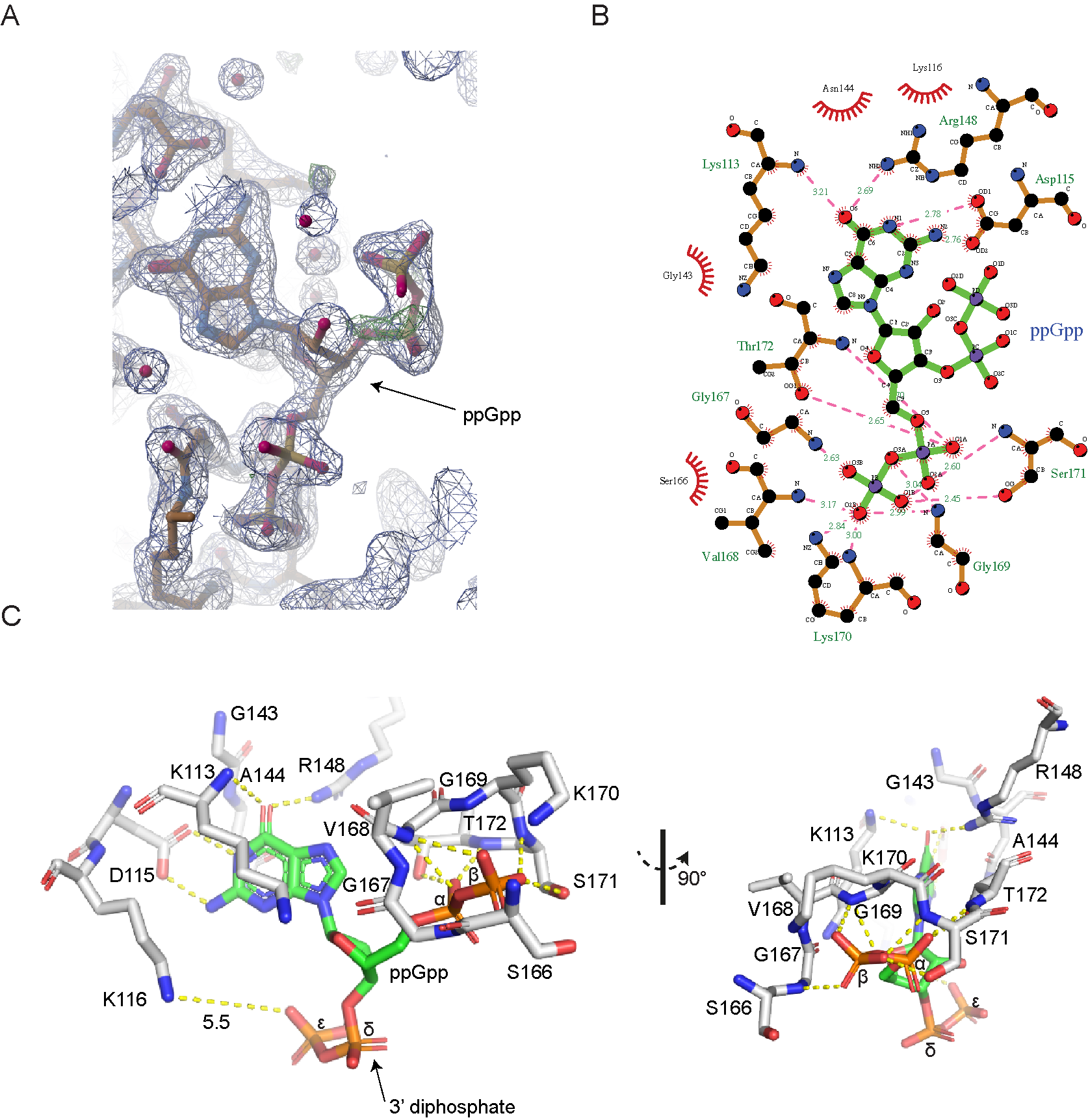

Supplement: FIG S6 [file mbio.02679-21-sf006.tif]

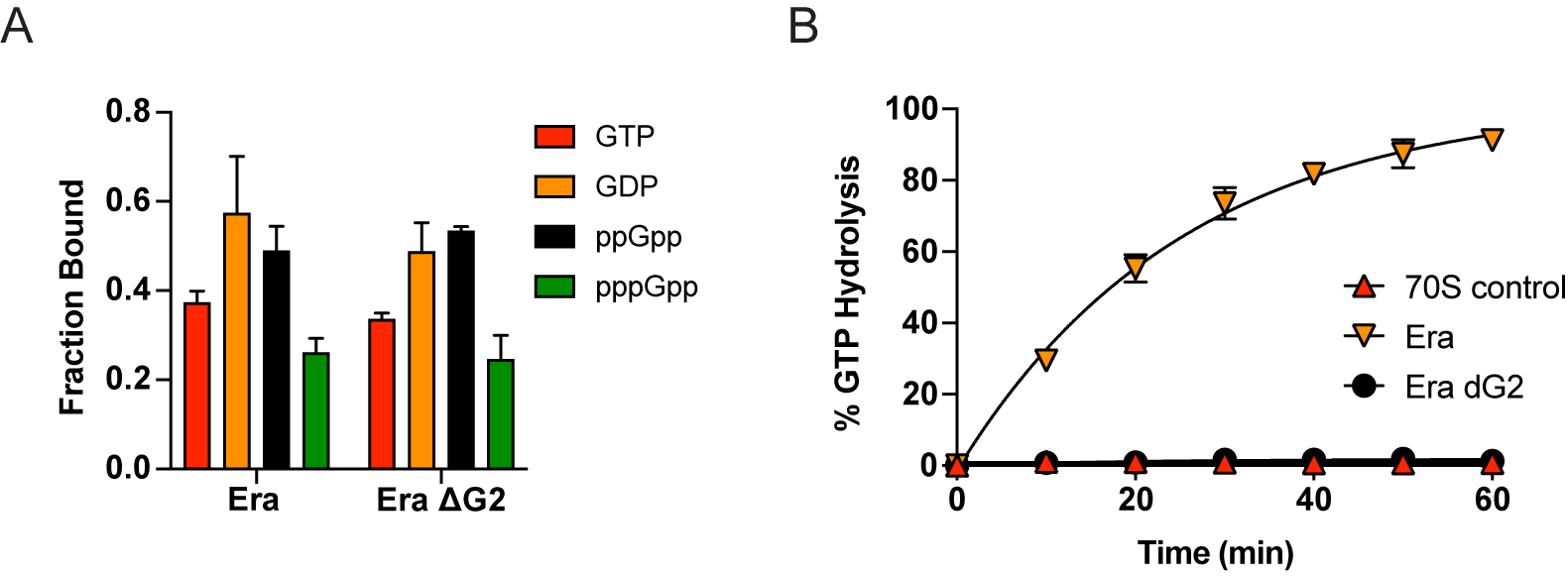

Supplement: FIG S7 [file mbio.02679-21-sf007.tif]
