# Supplementary material for: The Stringent Response Inhibits 70S Ribosome Formation in Staphylococcus aureus by Impeding GTPase-Ribosome Interactions
Source: mBio. 2021 Nov 9;12(6):e02679-21. doi: 10.1128/mBio.02679-21 (PMC8579695; doi:10.1128/mBio.02679-21)
Supplement: TABLE S2 [file mbio.02679-21-st002.docx]

**Table S2. Crystallographic data and refinement statistics**

|  | **RsgA Apo** | **RsgA-ppGpp** |
| --- | --- | --- |
| **Crystal data** |  |  |
| Space Group | P 1 2_1_ 1 | P 2_1_ 2_1_ 2_1_ |
| Unit Cell Dimensions (a, b, c (Å)) | 54.67   93.53   68.18 | 50.47   66.93  114.12 |
| Unit Cell Dimensions (α, β, γ (°)) | 90.00   90.72   90.00 | 90.00   90.00   90.00 |
|  |  |  |
| **Data Collection** |  |  |
| Wavelength (Å) | 0.97949 | 0.97949 |
| Resolution (Å) | 47.20-2.01 (2.06-2.01) | 57.73-1.94 (1.99-1.94) |
| Reflections (measure d/unique) | 306,023 | 375,925 |
| R_meas_ (%) | 0.149 (0.895) | 0.165 (0.990) |
| R_p.i.m._ (%) | 0.057 (0.336) | 0.063 (0.381) |
| <I/σI> | 8.4 (1.8) | 11.0 (2.5) |
| Multiplicity | 6.8 (7.0) | 12.8 (12.7) |
| Completeness (%) | 98.2 (97.0) | 99.9 (99.5) |
|  |  |  |
| **Refinement Statistics** |  |  |
| R_work_/R_free_ (%) | 22.41/27.83 | 21.63/26.22 |
| Average B factor (Å^2^) protein | 38.423 | 29.398 |
| Average B factor (Å^2^) solvent | 42.832 | 34.955 |
| Rmsd bond lengths (Å) | 0.0083 | 0.0096 |
| Rmsd bond angle (°) | 1.5890 | 1.8108 |
| Protein residues | 533 | 268 |
| Water molecules | 212 | 81 |
| Ions | 5 | 1 |
| Ramachandran (Favoured/Generous/Disallowed) | 494/27/4 | 255/7/1 |

Outer shell data in parenthesis. R_work_ = $\frac{\Sigma\left| \left| F_{obs} \right|-\left| F_{calc} \right| \right|}{\Sigma|F_{obs}|}$, where F_obs_ and F_calc_ are the observed and calculated factorial amplitudes of the structure respectively. R_free_ is calculated as above, except for a random subsection of data that was withheld from refinement. Ramachandran plot calculated within *Coot*. B factors calculated using Baverage within the CCP4 suite. Refinement statistics were read from the output log following crystallographic refinement via RefMac5 within the CCP4 suite (1, 2).

References

1. Emsley P, Lohkamp B, Scott WG, Cowtan K. 2010. Features and development of Coot. Acta Crystallogr D Biol Crystallogr 66:486-501.

2. Murshudov GN, Vagin AA, Dodson EJ. 1997. Refinement of macromolecular structures by the maximum-likelihood method. Acta Crystallogr D Biol Crystallogr 53:240-255.
