# Supplementary material for: The Stringent Response Inhibits 70S Ribosome Formation in Staphylococcus aureus by Impeding GTPase-Ribosome Interactions
Source: mBio. 2021 Nov 9;12(6):e02679-21. doi: 10.1128/mBio.02679-21 (PMC8579695; doi:10.1128/mBio.02679-21)
Supplement: TABLE S1 [file mbio.02679-21-st001.docx]

**Table S1. Bacterial strains used in this study**

| **Strain** | **Relevant features** | **Reference** |
| --- | --- | --- |
|  | ***Escherichia coli* strains** |  |
| XL1-Blue | Cloning strain: TetR | Stratagene |
| BL21(DE3) | Strain used for protein expression | Novagen |
| RMC0139 | pALC2073 in XL1-Blue: CarbR | (1) |
| RMC0147 | pET28b in XL1-Blue: KanR | Novagen |
| RMC0169 | pET28b-*gppA* in BL21(DE3): KanR | (2) |
| RMC0178 | pET21a-*relseq* in BL21(DE3): CarbR | (3) |
| RMC0399 | pET28b-*rsgA* in BL21 (DE3): KanR | (2) |
| RMC0401 | pET28b-*rbgA* in BL21 (DE3): KanR | (2) |
| RMC0402 | pET28b-*era* in BL21 (DE3): KanR | (2) |
| RMC0403 | pET28b-*hflX* in BL21 (DE3): KanR | (2) |
| RMC0531 | pCN55iTET (iTET) in XL1-Blue: CarbR | (4) |
| RMC0545 | pALC2073-*relP* in XL1-Blue: CarbR | This study |
| RMC1323 | pCN55iTET-*era*-his (iTET-*era*) in XL1-Blue: CarbR | This study |
| RMC1686 | pET28b-*era* ΔG2 in XL1-blue: KanR | This study |
|  |  |  |
|  | ***Staphylococcus aureus* strains** |  |
| SEJ1 | RN4220 *spa;* protein A negative derivative of RN4220; ANG314 | (5) |
| USA300 JE2 | CA-MRSA USA300 strain LAC derivative, lacking plasmids p01 and p03. Erm sensitive | (6) |
| USA300 JE2 (p)ppGpp-null | JE2 with in-frame deletions in *relQ*, *relP* and *rel:* ((p)ppGpp-null) | Gift from T. Stinear |
| USA300 LAC* | CA-MRSA USA300 strain LAC derivative, lacking plasmid p03. Erm sensitive | (7) |
| RMC0562 | USA300 LAC* iTET: SpecR | (4) |
| RMC0650 | USA300 LAC* Δ*era*: TetR | (4) |
| RMC0813 | USA300 LAC* Δ*era* iTET: TetR, SpecR | (4) |
| RMC1690 | USA300 LAC* Δ*era* iTET-*era*-his: TetR, SpecR | This study |
| RMC1847 | USA300 JE2 Δ*era* iTET-*era*-His: TetR, SpecR | This study |
| RMC1849 | USA300 JE2 Δ*rel* Δ*relP* Δ*relQ* Δ*era* iTET-*era*-His: TetR, SpecR | This study |
| RMC1865 | USA300 JE2 Δ*era* iTET-*era*-His pALC2073: TetR, CamR, SpecR | This study |
| RMC1866 | USA300 JE2 Δ*era* iTET-*era*-His pALC2073-*relP*: TetR, CamR, SpecR | This study |
|  |  |  |

Antibiotics were used at the following concentrations: for *E. coli* cultures: Kanamycin (KanR) 30 μg/ml, Carbenicillin (CarbR) 50 μg/ml; for *S. aureus* cultures: Tetracycline (TetR) 2 μg/ml, Chloramphenicol 7.5 μg/ml, Spectinomycin (SpecR) 250 μg/ml; IPTG at 1 mM, and anhydrotetracycline (Atet) at 100 ng/ml.

References

1. Bateman BT, Donegan NP, Jarry TM, Palma M, Cheung AL. 2001. Evaluation of a tetracycline-inducible promoter in *Staphylococcus aureus* in vitro and in vivo and its application in demonstrating the role of *sigB* in microcolony formation. Infect Immun 69:7851-7852.

2. Corrigan RM, Bellows LE, Wood A, Gründling A. 2016. ppGpp negatively impacts ribosome assembly affecting growth and antimicrobial tolerance in Gram-positive bacteria. Proc Natl Acad Sci USA 113:E1710-E1719.

3. Mechold U, Potrykus K, Murphy H, Murakami KS, Cashel M. 2013. Differential regulation by ppGpp versus pppGpp in *Escherichia coli*. Nucleic Acids Res 41:6175-6189.

4. Wood A, Irving SE, Bennison DJ, Corrigan RM. 2019. The (p)ppGpp-binding GTPase Era promotes rRNA processing and cold adaptation in *Staphylococcus aureus*. PLoS Genetics 15:e1008346.

5. Grundling A, Schneewind O. 2007. Genes required for glycolipid synthesis and lipoteichoic acid anchoring in *Staphylococcus aureus*. J Bacteriol 189:2521-2530.

6. Fey PD, Endres JL, Yajjala VK, Widhelm TJ, Boissy RJ, Bose JL, Bayles KW. 2013. A genetic resource for rapid and comprehensive phenotype screening of nonessential *Staphylococcus aureus* genes. mBio 4:e00537-12.

7. Boles BR, Thoendel M, Roth AJ, Horswill AR. 2010. Identification of genes involved in polysaccharide-independent *Staphylococcus aureus* biofilm formation. PLoS One 5:e10146.
